# Long noncoding RNA lncGALM increases risk of liver metastasis in gallbladder cancer through facilitating N‐cadherin and IL‐1β‐dependent liver arrest and tumor extravasation

**DOI:** 10.1002/ctm2.201

**Published:** 2020-11-10

**Authors:** Huaifeng Li, Yunping Hu, Yunpeng Jin, Yidi Zhu, Yajuan Hao, Fatao Liu, Yang Yang, Guoqiang Li, Xiaoling Song, Yuanyuan Ye, Shanshan Xiang, Yuan Gao, Jinhui Zhu, Yijian Zhang, Lin Jiang, Wen Huang, Jian Zhu, Xiangsong Wu, Yingbin Liu

**Affiliations:** ^1^ Department of General Surgery, Xinhua Hospital Affiliated to Shanghai Jiao Tong University School of Medicine Shanghai China; ^2^ Department of Biliary‐Pancreatic Surgery Renji Hospital Affiliated to Shanghai Jiao Tong University School of Medicine Shanghai China; ^3^ Shanghai Key Laboratory of Biliary Tract Disease Research Shanghai China; ^4^ Shanghai Research Center of Biliary Tract Disease Shanghai China; ^5^ State Key Laboratory of Oncogenes and Related Genes Shanghai China; ^6^ Department of General Surgery and Laparoscopic Center The Second Affiliated Hospital Zhejiang University School of Medicine Hangzhou China

**Keywords:** gallbladder cancer, interleukin 1β, liver sinusoidal endothelial cells, lncRNA‐lncGALM, miR‐200

## Abstract

**Background:**

Long noncoding RNAs (lncRNA) represent significant factors of the mammalian transcriptome that mediates varied biological and pathological processes. The liver is the most common site for gallbladder cancer (GBC) distant metastasis and contributes to the majority of GBC‐related death. How lncRNA affects GBC metastasis is not completely understood.

**Results:**

A novel lncRNA termed lncGALM (lncRNA in GBC associated with liver metastasis) was discovered to be highly expressed in cancer patients and xenografted tumors with liver metastasis. Elevated lncGALM in GBC patients also correlated to decreased survival. Invasion and migration of GBC cells were enhanced through lncGALM, both in vitro and in vivo. lncGALM functioned as sponges by competitively binding to and inactivating miR‐200 family members, which increase epithelial‐mesenchymal transition‐associated transcription factor ZEB1 and ZEB2, leading to a fibroblastic phenotype and increased expression of N‐cadherin. In addition, lncGALM bound to IL‐1β mRNA and stabilized the IL‐1β gene that mediates liver sinusoidal endothelial cell (LSECs) apoptosis. lncGALM‐expressing LiM2‐NOZ cells acquired a strong ability to migrate and adhere to LSECs, promoting LSECs apoptosis and therefore facilitating tumor cell extravasation and dissemination.

**Conclusions:**

lncGALM promotes GBC liver metastasis by facilitating GBC cell migration, invasion, liver arrest, and extravasation via the invasion‐metastasis cascade. Targeting lncGALM may be protective against the development of liver metastasis in GBC patients.

## Graphical abstract

(1) This study documents a novel lncRNA‐lncGALM from the lncRNA expression profiles of human GBC samples and animal models. (2) lncGALM promotes EMT in GBC cells by binding to miR‐200 family members. (3) lncGALM facilitates extravasation and liver colonization of GBC cells. (4) lncGALM could serve as a therapeutic target of GBC.

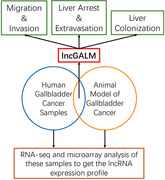

**Abbreviations**
ceRNA: competing endogenous RNA
EMT: epithelial‐mesenchymal transition
FISH: fluorescence in situ hybridization
GBC: gallbladder cancer
GFP: green fluorescent protein
IL1RA: interleukin‐1 receptor antagonist
IL‐1β: interleukin‐1β
LiM2: second round liver metastasis
lncGALM: lncRNA in GBC Associated with Liver Metastasis
lncRNA: long noncoding RNA
LSECs: liver sinusoidal endothelial cells
mRNA: messenger RNA
qRT‐PCR: quantitative real‐time polymerase chain reaction
RACE: rapid amplification of cDNA ends
RIP: RNA immunoprecipitation
UTR: untranslated region

## 1 BACKGROUND

Of all biliary tree related malignancies, gallbladder cancer (GBC) is the commonest. Liver dissemination is frequently encountered in this disease.^1^, ^2^, ^3^, ^4^ Over 90% of GBC patients who develop liver metastasis are inoperable and succumb quickly to their condition.^5^, ^6^, ^7^ Surgical resection is currently the only effective method expected to effectively ameliorate the disease; however, it is one that is extremely challenging.^5^, ^6^, ^7^ GBC carries with it a very poor prognosis, with a median survival time of less than 1 year.^8^, ^9^, ^10^ Identification of the molecular mechanisms behind the development of GBC is critical in developing more effective and less invasive treatment.

Long noncoding RNAs (lncRNAs) are a highly heterogeneous class of RNA molecules consisting of more than 200 nucleotides that are not able to encode for proteins.^11^ Nevertheless, these molecules are highly similar to messenger RNAs (mRNAs) in the fact that they are able to be polyadenylated, spliced, capped, and are transcribed by RNA polymerase II. lncRNAs are ubiquitous molecules and can be found within introns, in intergenic DNA, or in antisense gene overlap, in which they are only detected at certain developmental stages or in specific tissues.^12^ To date, lncRNAs have been found to mediate a diverse variety of cellular processes predominately through regulating gene expression of subcellular compartment organization, metabolism, cell survival and migration, as well as the cell cycle itself.^13^, ^14^ In cancer, while evidence implicates lncRNAs in several gastrointestinal cancer metastasis including hepatocellular carcinoma, colorectal cancer, and gastric cancer,^15^, ^16^, ^17^, ^18^, ^19^ lncRNAs that participate in GBC metastasis are poorly clarified. Therefore, to gain a deeper insight into the pathological role of potential lncRNAs in GBC liver metastasis, GBC patients with liver metastasis were selected and screened for the presence of a novel lncRNA, ENST00000522718 (NR_121620.1), named lncRNA in GBC Associated with Liver Metastasis (lncGALM). lncGALM has been documented to regulate epithelial‐mesenchymal transition (EMT)‐associated genes ZEB1 and ZEB2 via specifically binding to and sequestering miR‐200 family members. Upregulated N‐cadherin was found to facilitate adhesion and arrest of metastatic tumor cells in the liver. In addition, lncGALM induced interleukin‐1β (IL‐1β) expression that mediates invasive cancer cell extravasation, highlighting its role in multiple‐step metastasis that involves tumor cell migration, adhesion, extravasation, and relocalization in the liver. The current findings may yield greatly promising clinical results for the diagnosis and may be a potential therapeutic target of GBC patients with liver metastasis.

## 2 METHODS

### 2.1 Specimens of human GBC

The Institutional Ethical Board of Xinhua Hospital reviewed and approved all protocols related to this human study, with all participants enrolled only after providing informed consent. All of recruited patients did not receive radiotherapy or chemotherapy before cholecystectomy. Nine GBC patients with liver metastasis and 80 patients without metastasis diagnosed between 2013 and 2018 were included in this investigation. Immunohistochemical analyses were done on formalin‐fixed and paraffin‐embedded harvested samples, which encompassed the primary growth, adjacent healthy tissue, and/or liver metastatic deposits. After carefully screening, only three cases met the criteria for subsequent RNA sequencing analyses.

### 2.2 Cell lines

The Shanghai Institute for Biological Science, Chinese Academy of Science (Shanghai, China) supplied the SGC‐996 and GBC‐SD cell lines, while the Health Science Research Resources Bank (Osaka, Japan) provided the EH‐GB1 and NOZ cells lines. SGC‐996 cells were cultured in RPMI 1640 medium (Gibco, Grand Island, NY), while NOZ, GBC‐SD, and EH‐GB1 cells were cultured in Dulbecco's modified Eagle medium (Gibco). All cells were cultured with media supplemented with 10% fetal bovine serum (Gibco) in a 5% CO_2_ humidified incubator (Thermo Fisher Scientific) at 37°C.

### 2.3 Animals

Animal studies protocols were reviewed and approved by the Institutional Animal Care and Use Committee of Xinhua Hospital, Shanghai Jiao Tong University School of Medicine, Shanghai, China. Four‐ to six‐week‐old male BALB/c nude mice raised under specific pathogen‐free conditions were obtained from the Shanghai Laboratory Animal Center of the Chinese Academy of Sciences, Shanghai, China. Subsequent experiment was carried out in accordance to guidelines for use of laboratory animals stipulated by our institution.

### 2.4 RNA‐sequencing analysis

Liquid nitrogen was used to suspend ground tissue samples before the extraction of total RNA using All Prep DNA/RNA/Protein Mini Kit (QIAGEN, Germany). The Qubit™ RNA HS Assay Kit (Invitrogen, Carlsbad, CA) enabled quantification of the total RNA. After removal of rRNA using the KAPA Stranded RNA‐Seq Kit with RiboErase (KAPA), strand specific lncRNA library were constructed. The library quantity and quality were detected by the Qubit™ dsDNA HS Assay Kit (Invitrogen), while the inserts of the library were detected by High Sensitivity DNA Chip using the Agilent 2100 Bioanalyzer system. The molarity of the library was detected by Step One Plus™ Real‐Time PCR System. RNA‐sequencing was carried out with the Novaseq6000 V4 PE150 (Illumina) and 20G clean data were measured. For quality control of the reads, the cutadapt (v1.11) software was used to delete joint and inferior quality sequences. HISAT2 (v2.1.0) software allowed for comparison of clean data to the reference human genome (hg19). RSeQC software was used to access the position of genome reads. Gene expression levels of the RNA‐sequencing data were determined with the Kallisto (v0.44.0) software. The gene differential expression analysis of different groups was analyzed by DESeq2 software. The RNA sequencing data were deposited in GEO (GSE132223).

### 2.5 Liver metastatic mouse model of GBC

NOZ and GBC‐SD cells in the logarithmic growth phase were trypsin‐digested, counted, before being suspended in normal saline at a concentration of 1 × 10^6^/100 μL. Five‐week‐old male BALB/c nude mice were used for intrasplenic injection. After being anesthetized by 50 μL of 3% pentobarbital, the mice were positioned in a right lateral recumbent position. A 1‐cm‐long abdominal incision was made at the left anterior axillary line under the costal margin, and then the spleen was brought out using flat forceps. After mixing the cells thoroughly, 100 μL of the cell suspension was inoculated with an insulin syringe into the free edge of the spleen. Then, the spleen was compressed for 2 min, followed by a splenectomy. After closing the abdomen, the mice were warmed under an incandescent lamp until they were awake. All the procedures were performed in a sterile environment. The IVIS@ Lumina II system (Caliper Life Sciences, Hopkinton, MA) was used to monitor for the development of metastasis. Luciferin (Gold Biotech) was diluted in normal saline at a concentration of 100 μg/μL; bioluminescence was measured 10 min after 80 μL of the luciferin solution was injected intraperitoneally.

### 2.6 Isolation of liver metastatic derivatives

Mice with liver metastases were euthanized, with resection of liver metastatic lesions performed via an aseptic technique. Harvested lesions were cubed into 1 mm^3^ pieces before being cultured under gentle agitation for 3‐4 hours at 37°C with DMEM/F12 (1:1) (GIBCO) supplemented with 0.125% collagenase IV. After the collagenase treatment, a 100 μm cell strainer (BD Falcon) was used for cell filtration, briefly centrifuged, and then resuspended for culture in DMEM.

### 2.7 Microarray analysis

Samples (three NOZ and three second round liver metastasis (LiM2)‐NOZ cells) were selected randomly for microarray analysis. Total RNA was extracted using the TRIzol Reagent (Invitrogen), with the NanoDrop ND‐1000 used to assess RNA quality and quantity. RNA integrity was assessed via a standard denaturing agarose gel electrophoresis. The Agilent One‐Color Microarray‐Based Gene Expression Analysis protocol (Agilent Technology) was used to perform sample array hybridization and sample labeling, with subsequent array images acquired via the Agilent Feature Extraction software (version 11.0.1.1). Quantile normalization and data processing were performed using the GeneSpring GX v12.1 software package (Agilent Technologies). Significantly differentially expressed mRNAs and lncRNAs between any two samples were first identified through fold‐change filtering and were assessed via *P* values and FDR filtering. Homemade scripts were used to carry out combined analyses and hierarchical clustering. The microarray data were deposited in GEO (GSE106671).

### 2.8 RNA extraction and qRT‐PCR

The TRIzol reagent (Invitrogen) was used to extract total RNA from fresh tissues prior to cDNA synthesis with the PrimeScript™ RT reagent Kit and gDNA Eraser (Takara). Real‐time PCR was performed using SYBR® Premix Ex Taq™ II (Takara). Endogenous controls for miRNA was U6 while lncRNA and mRNA expressions were compared against GAPDH. Table S5 depicts quantitative real‐time polymerase chain reaction (qRT‐PCR) primers.

### 2.9 5′ and 3′ Rapid amplification of cDNA ends (5′ 3′ RACE)

A TRIzol Plus RNA Purification Kit (Invitrogen) was used to extract total RNA following protocols stipulated by the manufacturer. Synthesis of 5′ and 3′ rapid amplification of cDNA ends (RACE) templates was then done with the GeneRacer™ Kit (Invitrogen). Table S5 depicts the primers used for 5′ and 3′ RACE.

### 2.10 Northern blot analysis

Northern blot analysis was performed as previously described. A TRIzol Plus RNA Purification Kit (Invitrogen) was used for total RNA extraction, which were then subjected to formaldehyde gel electrophoresis. Then, the RNA was blotted onto a Biodyne Nylon membrane (Pall, NY) for 8 hours before being cross‐linked in a UV cross‐linker. The membrane was prehybridized overnight at 60°C in an ULTRAhyb buffer (Ambion, Grand Island, NY) before being hybridized a second time overnight at 60°C with the same ULTRAhyb buffer solution but with the addition of biotin‐labeled probe. Samples were then rinsed and blocked prior to analysis of lncGALM expression. Table S5 depicts all probe sequences.

### 2.11 Fluorescence in situ hybridization

Fluorescence in situ hybridization (FISH) was performed as previously described.^8^ The probe used for lncGALM is listed in Table S5.

### 2.12 Nuclear and cytoplasmic RNA isolation

RNA from cell nucleus and cytoplasm was extracted from NOZ cells and processed with the PARIS™ Kit (Invitrogen) based on protocols according to manufacturer's instruction.

### 2.13 In vitro translation

The TNT® T7 Quick Coupled Transcription/Translation Systems and Transcend™ Non‐Radioactive Translation Detection Systems (Promega) kit was used for in vitro translation assay based on manufacturer’ protocols.

### 2.14 Vector construction

The cDNA encoding lncGALM, lncGALM with miR‐200 binding site point mutations (GCAGGATT mutated to TACCCTGA, ACAGCGTT mutated to TATCACGA), and lncGALM with IL‐1β binding site point mutations (binding site deletion) were produced using GenScript (Nanjing, China) and cloned into the Hind III and EcoR I site of pcDNA3.1(+) vectors (Invitrogen), named pcDNA3.1‐lncGALM, pcDNA3.1‐lncGALM‐mut(miR‐200), and pcDNA3.1‐lncGALM‐mut(IL‐1β), respectively. pSL‐MS2‐12X (Addgene) was double digested with EcoR I and Xho I, and the MS2‐12X fragment was cloned into pcDNA3.1, pcDNA3.1‐lncGALM, pcDNA3.1‐lncGALM‐mut(miR‐200), and pcDNA3.1‐lncGALM‐mut(IL‐1β), yielding pcDNA3.1‐MS2, pcDNA3.1‐MS2‐lncGALM, pcDNA3.1‐MS2‐lncGALM‐mut(miR‐200), and pcDNA3.1‐MS2‐lncGALM‐mut(IL‐1β). pcDNA3.1‐lncGALM, pcDNA3.1‐lncGALM‐mut(miR‐200), and pcDNA3.1‐lncGALM‐mut(IL‐1β) were double digested with Hind III and EcoR I, and the lncRNA fragment was cloned into pBluescript II SK (+), yielding pBluescript II SK‐lncGALM, pBluescript II SK‐lncGALM‐mut(miR‐200), and pBluescript II SK‐lncGALM‐mut (IL‐1β). The 3′ 500 nt of lncGALM, lncGALM‐mut(miR‐200), and 3′untranslated region (UTR) of ZEB1/ZEB2 underwent PCR amplification prior to being cloned into the pmirGLO vector (Promega, Madison, WI) for use in the luciferase reporter assay.

### 2.15 Stable cell line generation via lncGALM overexpression or knockdown

In order to generate cells that had stable expressions of lncGALM, lncGALM‐mut(miR‐200), lncGALM‐mut(IL‐1β), NOZ, and GBC‐SD cells were transfected with pcDNA3.1‐lncGALM, pcDNA3.1‐lncGALM‐mut(miR‐200), and pcDNA3.1‐lncGALM‐mut(IL‐1β) with the help of Lipofectamine™ 2000 Transfection Reagent (Invitrogen). G418 (2 mg/mL) was used to select cells that underwent a 48‐hour transfection period over 4 weeks.

Synthesis of shRNA‐lncGALM, shRNA‐IL‐1β, and negative control shRNA was done prior to their insertion into the hU6‐MCS‐Ubiquitin‐EGFP‐IRES‐puromycin lentiviral vector (Genechem, Shanghai, China). To obtain cell lines stably expressing lncGALM or IL‐1β shRNA, LiM2‐NOZ and LiM2‐GBC cells were transfected with these recombinant lentiviruses. After 48 hours, the cells were selected with puromycin (1 μg/mL for LiM2‐NOZ and 3.5 μg/mL for LiM2‐GBC) for 2 weeks. Table S6 demonstrates shRNA sequences utilized in this investigation.

### 2.16 Transient transfection

Plasmids, miRNA mimics, and inhibitors were transiently transfected with Lipofectamine™ 2000 Transfection Reagent (Invitrogen); the final concentration of the miRNA mimics and inhibitors was 100 nM. siRNAs were transfected with 100 nM of Rfect siRNA Transfection Reagent (BIOGEN, Changzhou, China). Table S6 depicts the siRNA sequences utilized in this study.

### 2.17 In vitro cell invasion and migration studies

The in vitro invasion and migration ability of GBC cells were assessed by transwell assay that utilized 8‐μm sized filters (BD Biosciences) with or without a Matrigel (BD Biosciences) coating. Chambers in the upper half were filled with 2 × 10^4^ each of EH‐GB1, SGC‐996, GBC‐SD, and NOZ cells in addition to 10% FBS‐supplemented media (700 μL) in the lower chamber. The cells were left for 18 hours of incubation in a humidified incubator, rinsed thrice by PBS, and subjected to a 20‐minute fixation period with 4% paraformaldehyde and subsequent 15‐minute staining with crystal violet. The cells were rinsed another three times with PBS before cotton swabs used to scrape away cells on the upper chamber. All experiments were repeated three times.

### 2.18 Cell proliferation analysis

Cell proliferation rates analyzed by cell‐counting kit 8 (CCK‐8; Dojindo Laboratories) assay according to the manufacturer's instructions. After 1 × 10^3^ cells were aliquotted onto plates consisting of 96 wells each, all wells received 10 μL of CCK8 at predetermined time points and incubated for 2 hours. The absorbance at 450 nm was measured as an indicator of cell proliferation. The experiments were performed in triplicate.

### 2.19 Dual‐luciferase reporter assay

PmirGLO, pmirGLO‐lncGALM, or pmirGLO‐lncGALM‐mut (miR‐200) vectors were cotransfected with miR‐200s by Lipofectamine™ 2000 Transfection Reagent (Invitrogen) in NOZ cells. Luciferase intensity was measured 24 hours later via the Dual‐Luciferase® Reporter Assay System (Promega), following guidelines from the manufacturer. Renilla luciferase intensity was used as the control.

### 2.20 RNA affinity pulldown assay

LncGALM, lncGALM‐mut (miR‐200), and lncGALM‐mut (IL‐1β) were transcribed in vitro following the pBluescript II SK‐lncGALM, pBluescript II SK‐lncGALM‐mut(miR‐200), and pBluescript II SK‐lncGALM‐mut (IL‐1β) by T7 RNA polymerase (Roche) and labeled with biotin using the Biotin RNA Labeling Mix (Roche). Purification of RNA products were performed with the RNeasy Mini Kit (Qiagen, Valencia, CA). In total, 3 μg of transcripts were suspended for 2 hours with 1 mg of whole cell lysates from NOZ cells at 25°C. Additionally, isolation of complexes was performed with the streptavidin‐coupled magnetic beads (Invitrogen). RNAs were extracted from the complexes and subjected to purification and qRT‐PCR analysis.

### 2.21 MS2‐RIP

NOZ cells were co‐transfected with pcDNA3.1‐MS2, pcDNA3.1‐MS2‐lncGALM, pcDNA3.1‐MS2‐ lncGALM‐mut(miR‐200), or pcDNA3.1‐MS2‐lncGALM‐mut(IL‐1β) with pMS2‐GFP (Addgene). Magna RNA immunoprecipitation (RIP) RNA‐Binding Protein Immunoprecipitation Kit (Millipore, Bedford, MA) was used to perform RIP after 48 hours utilizing the green fluorescent protein (GFP) antibody based on protocols set by the manufacturer. RNAs extracted from the protein‐RNA complexes were analyzed by qRT‐PCR.

### 2.22 Western blot analysis

Previously illustrated protocols for Western blot analysis were utilized.^8^ RIPA buffer (Beyotime, Shanghai, China) was first used to lyse GBC cells on ice. Samples of proteins of approximately the same amount were electrophoresed using a 10% polyacrylamide gel before being blotted onto a PVDF membrane (Millipore, Darmstadt, Germany). Samples were then subjected to a 1‐hour blocking period with 5% skim milk before an overnight incubation with indicated primary antibodies at 4°C, followed by incubation with second antibody for 30 minutes (1:5000; Abcam, Cambridge, UK). Membranes were imaged with an ECL detection reagent (Rockford, IL). The primary antibodies are listed in Table S7.

### 2.23 LSECs isolation

Male BALB/c nude mice were euthanized and then sterilized with 75% alcohol. All the procedures were performed under aseptic conditions. After the livers were excised and rinsed with PBS, they were cut into 1 mm^3^ pieces and incubated with 2 mg/mL Dispase II (Roche) at 4°C overnight. The tissue pieces were then squeezed with forceps for adequate liver sinusoidal endothelial cell (LSEC) release. Cell filtration was done using a 100‐μm cell strainer (BD Falcon) before being centrifuged for 5 minutes at 2000 rpm. Cells were then resuspended in DMEM/F12 (1:1) (GIBCO). Then, the cells were separated with 35% Percoll (Pharmacia) using a density gradient centrifugation method. After centrifuging at 1800 rpm for 10 min, cells between the red blood cells and Percoll were collected, rinsed with DMEM/F12 (1:1), and then cultured in endothelial cell medium (ECM; ScienCell) for 12 hours, after which the cells were rinsed by PBS. After 3 days, the cells were digested with 0.05% trypsin (GIBCO). When the cells began to contract, the digestion was stopped immediately and the cells were rinsed twice using PBS. ECM was then used for further cell culture. This purification procedure was performed two or three times until almost all the nonparenchymal cells were removed. Immunofluorescence for von Willebrand factor (vWF) and CD31 were used for purity estimation.

### 2.24 Immunofluorescence

Coverslips were placed into wells of 24‐well plates before the seeding of cells on top of them. These coverslips were then rinsed thrice with PBS before being fixed for 20 minutes with 4% paraformaldehyde and permeabilized for 10 minutes with 0.5% Triton X‐100. PBS was then used to rinse the samples thrice before they were exposed for 1 hour to 1% BSA at room temperature. Primary antibodies were added to the samples for an overnight incubation at 4°C. The cells were washed with PBS thrice and incubated for 30 minutes with FITC‐conjugated goat antibodies against rabbit IgG (Abcam, Cambridge, UK) before being subjected to another three washes with PBS. Nuclei counterstaining was done with a 10‐second exposure to 4′,6‐diamidino‐2‐phenylindole (Invitrogen) before undergoing three final rinses with PBS. All coverslips were imaged by fluorescence microscopy (Leica). The primary antibodies are listed in Table S7.

### 2.25 Scanning electron microscope

Mice were anesthetized by 50 μL of 3% pentobarbital and fixed at supine position. The abdomen was opened and the portal vein was separated. We perfused the liver via the portal vein with 15 mL normal saline using a 24G venous indwelling needle, followed by 5 mL 2.5% glutaraldehyde perfusion. Note that 1 mm^3^ liver was excised using a scalpel while avoiding traction and extrusion before being rinsed with PBS. Blocks of tissue were fixed in 2.5% glutaraldehyde followed by 1% osmic acid. Tissues underwent serial ethanol dehydration with 30%, 50%, 70%, 80%, 90%, 95%, 100%, and 100% ethyl alcohol successively before being dried in critical point dryer and sputtered with gold in an ion sputter coater. The tissues were observed under a scanning electron microscope (HITACHI, Japan).

### 2.26 LSECs permeability assay

The permeability assay was carried out based on past guidelines.^20^ In short, LSECs were seeded on 0.4 μm transwell filters (Greiner Bio‐One) in a 24‐well plate and cultured for 12 hours. Different conditioned mediums with 0.5 μg/μL 40 kDa FITC‐Dextran (Sigma Aldrich) was added in the upper chamber and dye concentration in the lower chamber was analyzed for excitation and emission at 492 nm and 520 nm on the ELISA reader after 24 hours.

### 2.27 TUNEL staining

Sections processed with formalin and embedded in paraffin were used for the TUNEL assay. One Step TUNEL Apoptosis Assay Kit was obtained from Beyotime and used to carry out the assay in strict compliance to instructions set by the manufacturer.

### 2.28 IL‐1β ELISA

The IL‐1β level in the cell culture supernatant was measured using a human IL‐1β ELISA Kit (ABclonal) according to the manufacturer's instructions.

### 2.29 Statistical analysis

The GraphPad Prism software was used to carry out all statistical analysis. The mean values of two groups were compared using the two‐tailed Student's *t*‐tests, with mean values of more than two groups analyzed using two‐way analysis of variance. The association of lncGALM expression with clinicopathological variables was analyzed by Pearson *χ* ^2^ tests. Kaplan‐Meier plots and log‐rank tests were used for the survival analysis. Where appropriate, we applied the two‐tailed Fisher's exact test. Statistical significance was achieved when *P* values were less than .05.

## 3 RESULTS

### 3.1 An upregulation of lncGALM correlated with poor clinical outcome in GBC liver metastatic tumors

**FIGURE 1:**
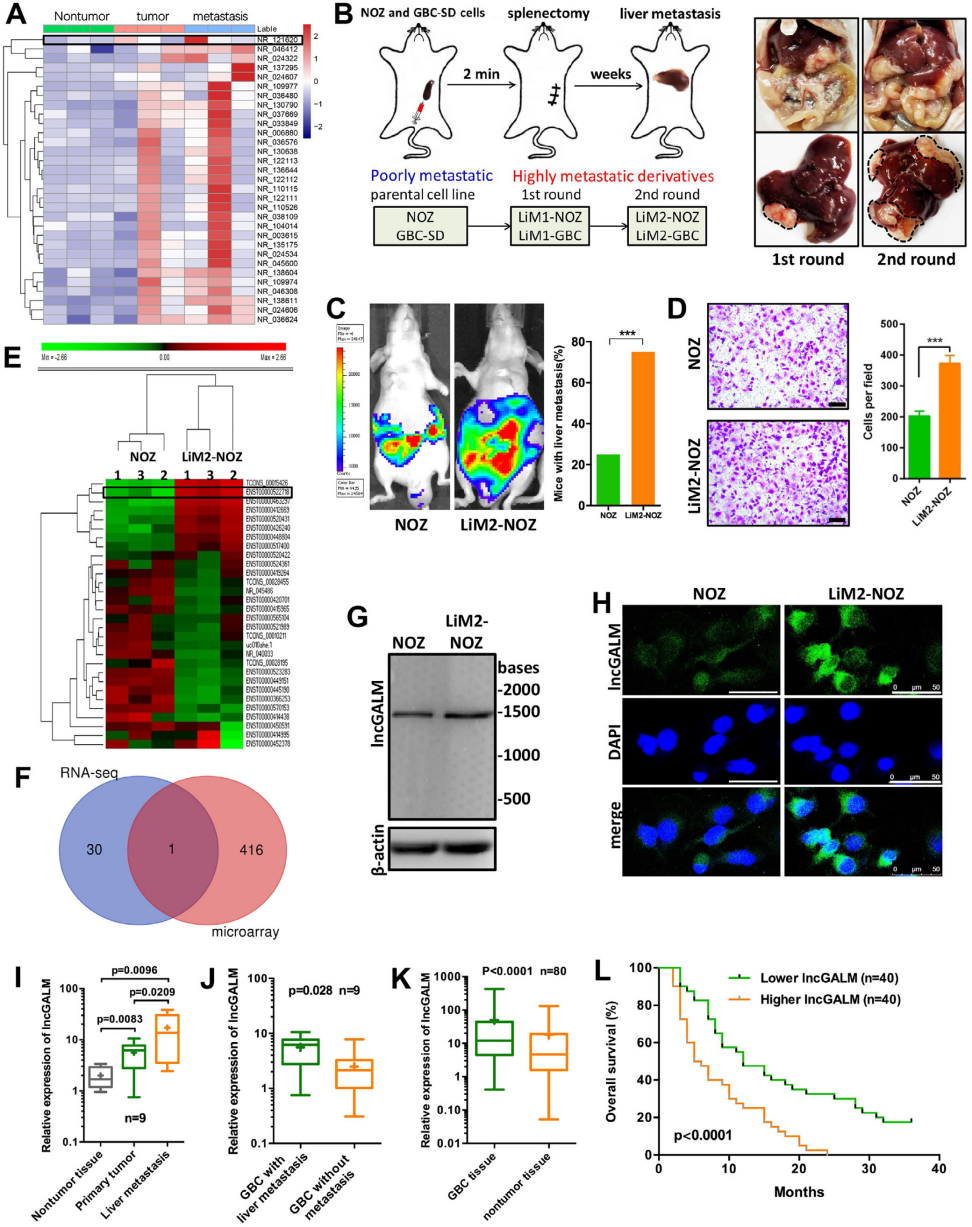
Upregulated lncGALM was found in GBC liver metastatic tumors and correlated with poor clinical outcome. **A,** RNA‐sequencing analysis in patient tissues including adjacent nontumor tissues, primary GBC tissues, and liver metastatic tumor tissues of three GBC patients. **B,** Scheme of the orthotopic xenograft mouse model of GBC liver metastasis(left); first and second round liver metastasis lesions (right). **C,** Liver metastasis rate of NOZ and LiM2‐NOZ cells, 20 mice were used per cell line, two‐tailed Fisher's exact test was used to calculate statistical significance. **D,** Migration of NOZ and LiM2‐NOZ cells was assessed by transwell assay (*n* = 3, scale bar, 100 μm) (right). **E,** Hierarchical clustering analysis of lncRNAs that highly expressed in LiM2‐NOZ cells compared with NOZ cells by microarray. **F**, Venn‐diagram of the microarray and RNA‐seq data. **G,** Northern blot analysis to confirm the size and expression of lncGALM. **H,** FISH was performed to detect the expression level and location of lncGALM in different cell clones (scale bar, 50 μm). **I,** Expression levels of lncGALM in GBC tissues, including primary tumor tissues, adjacent nontumor tissues and liver metastatic tumor tissues of nine patients. **J,** LncGALM was highly expressed in primary tumors with liver metastasis compared with tumors without metastasis (n = 9). **K,** LncGALM was highly expressed in tumor tissues compared with paired adjacent nontumor tissues (n = 80). For (H)‐(J), the expression level of lncGALM was analyzed by qRT‐PCR, the horizontal lines in the box plots represent the median, the boxes represent the interquartile range, the whiskers represent the minimum and maximum values, and the mean values were indicated by “+.” **L,** Kaplan‐Meier analysis of the correlation between lncGALM expression and the overall survival of 80 patients with GBC. The median expression level was used as the cutoff (***P < .001)

To determine if there were some lncRNAs potentially associated with GBC liver metastasis, we began screening liver metastasis tumor samples. Even though the population of patients with liver metastasis was quite small, we fortunately identified three tumor samples from nine cases that met our criteria for the study and contained adjacent benign tissue, primary tumor, and liver metastatic tumor. As demonstrated in Figure 1A and Table S1, by RNA sequencing analysis, 31 lncRNA candidates were differentially expressed in these three samples, with an increasing trend of expression from nontumor to metastatic tumor samples. We chose three lncRNAs randomly and validated the RNA‐seq data by qRT‐PCT in nine patients (Figure S1A). To firmly establish that at least some of these lncRNAs play an essential role in the liver metastasis virtually, we utilized a complementary approach by means of developing a mouse model of GBC with liver metastasis. This murine model was created by splenic administrations with GBC cell lines NOZ and GBC‐SD (Figure 1B and Figure S1B and C). Following two continuous rounds of transplenic metastasis implantation, we successfully obtained highly invasive cell lines from each parental line, named LiM2‐NOZ and LiM2‐GBC, respectively. Both LiM2‐NOZ and LiM2‐GBC demonstrated the stronger ability to metastasize to the liver in vivo and invade transwell membrane in vitro than their parental cells (Figure 1C and D and Figure S1D and E). Consequently, the lncRNA microarray analysis revealed that a total of 417 lncRNAs were upregulated by at least threefold higher in LiM2‐NOZ than in NOZ cells (Figure 1E, Table S2). In those differentially expressed transcripts, we were particularly interested in one candidate ENST00000522718 (NR_121620.1) that was also similarly upregulated in human tumor samples (**Figure** **1** **F**). This candidate was hence named lncGALM. LncGALM is located on chromosome 8 in *Homo sapiens* and is composed of six exons with a full length of 1471 nt, as confirmed using rapid cDNA amplification using 5′ and 3′ ends (termed the RACE assay) (Figure S1F and G). Northern blot analysis correlated to the above findings, showing high expression levels of lncGALM in LiM2‐NOZ cells in contrast to parental cells (Figure 1G). Consistent with these data, FISH showed higher cytoplasm levels of lncGALM in LiM2‐NOZ cells than those in NOZ (Figure 1H). Finally, the noncoding nature of lncGALM was confirmed by the ORF Finder, a coding potential analyzer constructed by the National Center for Biotechnology Information (Figure S1H) and the Coding Potential Calculator (CPC) (Figure S1I), both of which failed to detect a protein built with 50 amino acids or more for lncGALM. In addition, the coding probability predicted using the Coding‐Potential Assessment Tool (CPAT) was only 0.018 (Figure S1J), which strongly suggests that lncGALM does not possess any potential to code for proteins. Meanwhile, an in vitro translation assay was done to prove that lncGALM cannot synthesize proteins (Figure S1K).

In evaluating lncGALM levels in GBC with liver metastasis, we examined the nine metastatic cases and found that lncGALM levels were higher in liver metastatic tumors than in primary tumors and adjacent nontumor tissue (Figure 1I). In addition, lncGALM levels in the primary tumors of cases demonstrating liver metastasis were elevated in contrast to those that did not have liver metastasis (Figure 1J). In 80 cases of GBC lacking distant metastasis, we found that tumor tissues had higher levels of lncGALM in contrast to surrounding healthy tissue (Figure 1K). The elevated levels of lncGALM in these 80 cases positively correlated with cells that were more poorly differentiated, the presence of lymph node metastasis, tumor invasion, and a higher TNM staging of GBC (Table 1). Furthermore, Kaplan‐Meier analysis revealed that an elevated lncGALM expression was associated with reduced overall survival (Figure 1L). Interpreted as a whole, our findings highlight the potential of lncGALM expressions as a useful tool in prognosticating GBC.

**TABLE 1:** Association of lncGALM expression levels and clinicopathological characteristics of 80 GBC patients

Characteristics	lncGALM		*P*
	High	Low	
All cases	40	40	
Age			
<60	12	10	.617
≥60	28	30	
Gender			
Male	16	14	.644
Female	24	26	
Gallstone			
Present	25	22	.496
Absent	15	18	
Tumor size			
<4 cm	15	22	.116
≥4 cm	25	18	
Histology differentiation			
Well or moderate	13	30	<.001^*^
Poor	27	10	
Tumor invasion (AJCC)			
Tis. ‐T2	3	33	<.001^*^
T3‐T4	37	7	
Lymph node metastasis			
Present	27	13	.002^*^
Absent	13	27	
TNM stage (AJCC)			
0‐II	0	26	<.001^*^
III‐IV	40	14	

^*^ *P* < 0.05

### 3.2 lncGALM induced GBC invasive activity both in vitro and in vivo

**FIGURE 2:**
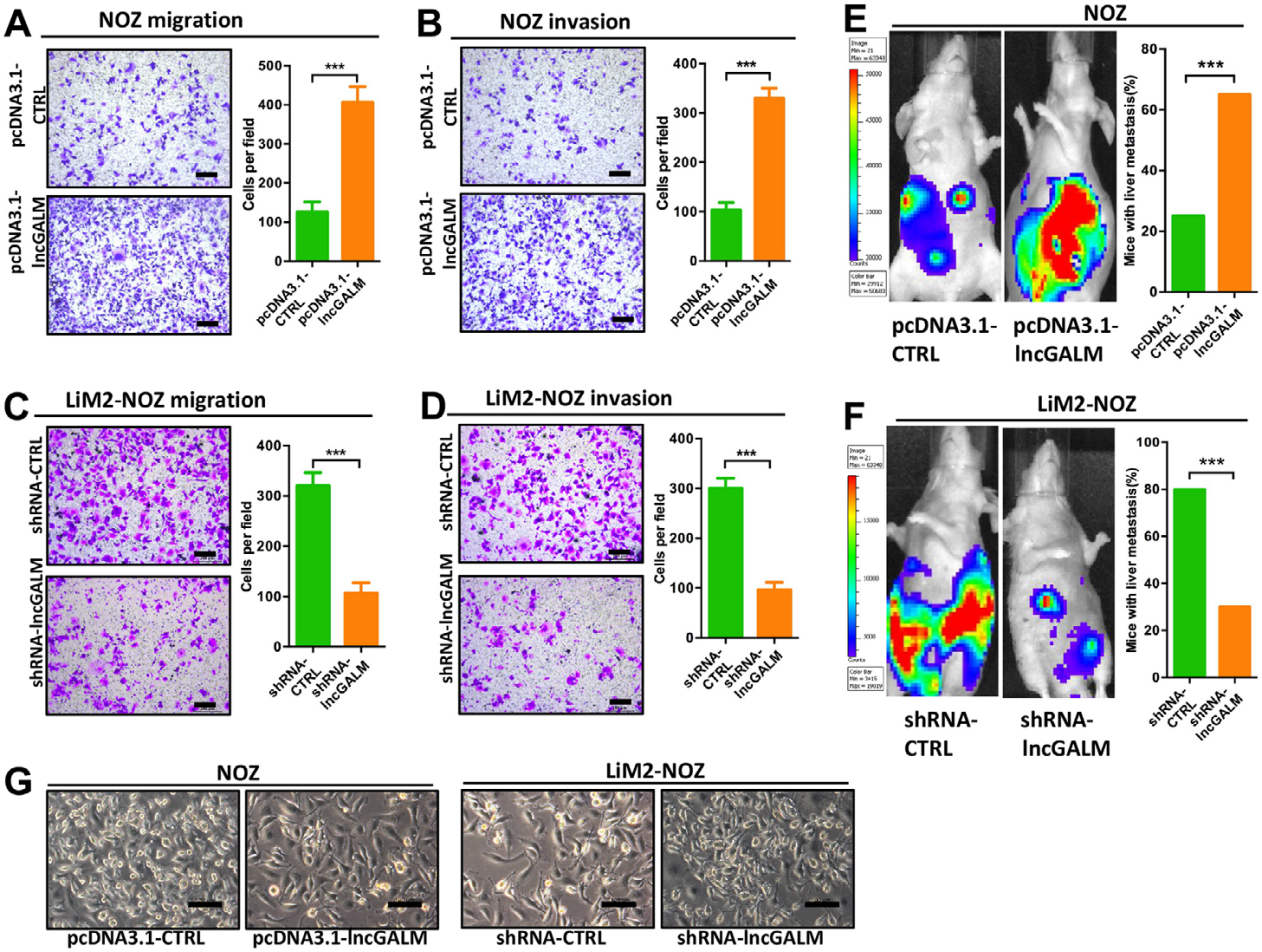
LncGALM induced GBC invasive activity both in vitro and in vivo. **A and B,** Overexpression of lncGALM significantly increased migration (A) and invasion (B) of NOZ cells in vitro. **C and D,** Knockdown of lncGALM significantly decreased migration (C) and invasion (D) of LiM2‐NOZ cells in vitro. **E,** Overexpression of lncGALM promoted NOZ cell liver metastasis. Twenty mice were used per cell line. **F,** Knockdown of lncGALM suppressed LiM2‐NOZ cell liver metastasis, and 20 mice were used per cell line. **G,** Overexpression of lncGALM induced mesenchymal‐like morphological features in NOZ cells, while the knockdown of lncGALM reversed this change in LiM2‐NOZ cells (scale bar, 100μm). (***P < .001)

Our studies sought to investigate the contribution of lncGALM in GBC metastasis. To achieve this, we stably overexpressed lncGALM in NOZ and GBC‐SD cells, but knocked down lncGALM in LiM2‐NOZ and LiM2‐GBC cells (Figure S2A). LncGALM overexpression induced higher cell invasiveness and migratory capabilities (three to four times higher) in contrast to normal NOZ and GBC‐SD cells (Figure 2A and B and Figure S2B and C), while lncGALM knockdown decreased cell invasion and migration to around 30% of controls (Figure 2C and D and Figure S2D and E). This altered expression of lncGALM in these cells did not influence cell proliferation (Figure S2F and G). In agreement with the observed in vitro findings, orthotopic xenografts displayed that the overexpression of lncGALM in NOZ and GBC‐SD cells promoted liver metastasis. lncGALM knockdown in LiM2‐NOZ and LiM2‐GBC cells suppressed liver metastasis (Figure 2E and F and Figure S2H and I). Of note, lncGALM‐overexpressed NOZ and GBC‐SD cells were of mesenchymal‐like morphology, but lncGALM knocked down LiM2‐NOZ and LiM2‐GBC cells showed an epithelial phenotype (Figure 2G and Figure S2J). Altering the expression of lncGALM promotes EMT in GBCs, the event which mediates tumor metastasis.

### 3.3 LncGALM induced EMT in GBC cells by binding to miR‐200 family members

**FIGURE 3:**
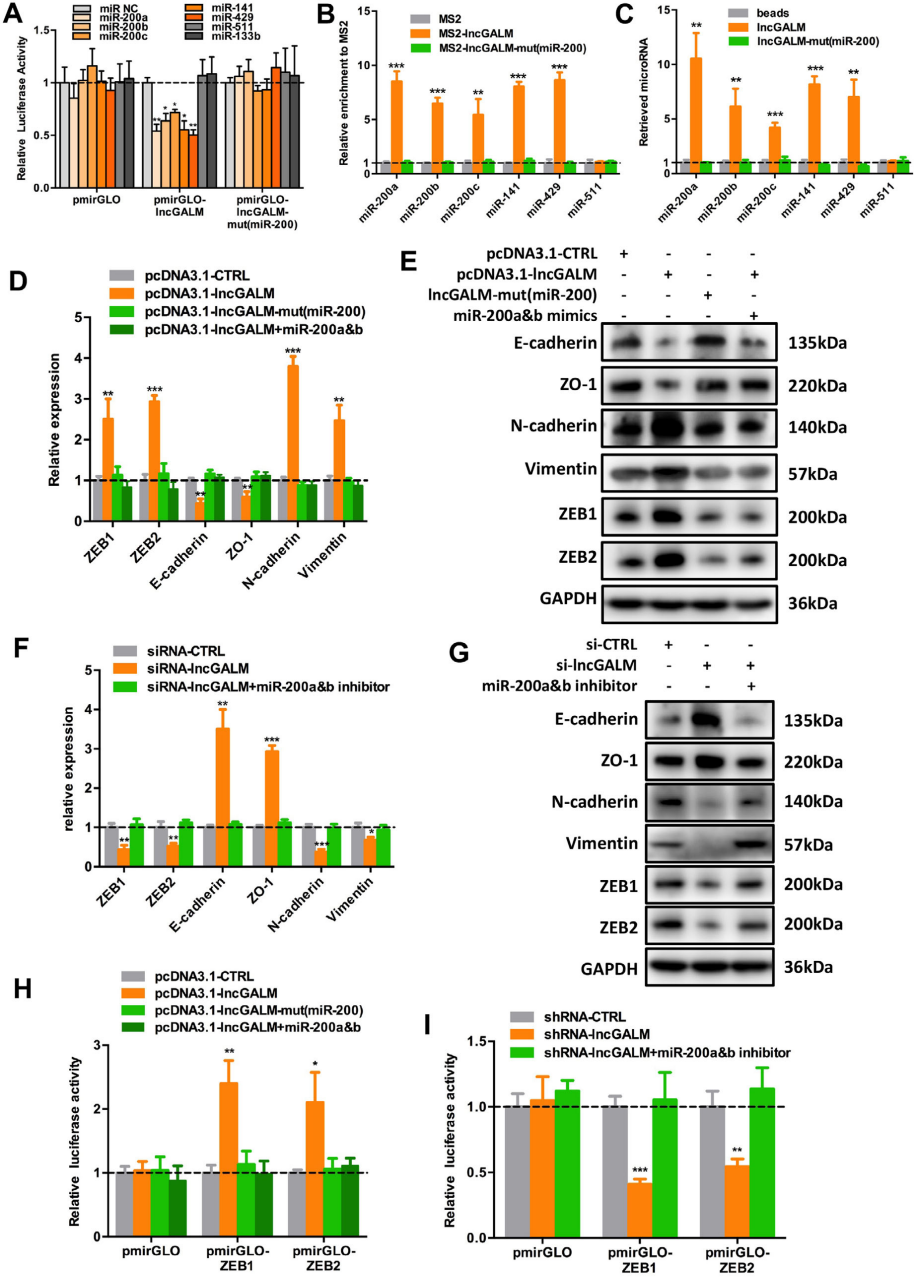
LncGALM induced EMT in GBC cells by binding to miR‐200 family members. **A,** Luciferase reporter assay in NOZ cells showed that miR‐200 family members bound to lncGALM and decreased luciferase activity; this effect was abolished by mutating the binding sites. Data were presented as the relative ratio of firefly luciferase activity to renilla luciferase activity. **B,** MS2‐RIP followed miRNA qRT‐PCR was used to test the endogenous association between lncGALM and the miR‐200 family. **C,** RNA affinity pulldown followed by miRNA qRT‐PCR was used to test the endogenous association between lncGALM and the miR‐200 family. **D and E,** mRNA (D) and protein (E) levels of ZEB1, ZEB2, and EMT markers in different NOZ cell clones after lncGALM overexpression. **F and G,** mRNA (F) and protein (G) levels of ZEB1, ZEB2, and EMT markers in different LiM2‐NOZ cell clones after lncGALM knockdown. **H,** Luciferase activity in different NOZ cell clones transfected with luciferase reporters containing ZEB1 3′UTR or ZEB2 3′UTR. **I,** Luciferase activity in differentLiM2‐NOZ cell clones transfected with luciferase reporters containing ZEB1 3′UTR or ZEB2 3′UTR. (*P < .05, **P < .01, ***P < .001)

Given that lncGALM is predominantly located in the cytoplasm (Figure 1G and Figure S3A), we anticipated its potential role as a competing endogenous RNA (ceRNA) that commonly acts as sponges to sequester specific miRNAs, resulting in release of miRNA binding from target genes.^15^, ^21^, ^22^, ^23^ To test this hypothesis, we ran the Segal Lab microRNA targets prediction software (https://genie.weizmann.ac.il/pubs/mir07/mir07_prediction.html) and predicted a putative miRNA group that may bind to the 3′ 500 nt of lncGALM (Table S3). We were particularly interested in the miR‐200 family that includes miR‐200a, miR‐200b, miR‐200c, miR‐141, and miR‐429, given its reported ability to suppress EMT and tumor metastasis by targeting the 3′ UTR of ZEB1 and ZEB2 mRNAs.^15^, ^24^, ^25^ Indeed, there were two miR‐200 binding sites present at the 3′ 500 nt of lncGALM in a relatively short span (Figure S3B and C), implicating that lncGALM may serve as a ceRNA for the miR‐200 family that inhibits ZEB1 and ZEB2 mRNAs. To validate that lncGALM binds to miR‐200s, first, we employed a pmirGLO luciferase reporter assay by constructing the 3′ 500 nt of lncGALM into the vector. Only miR‐200s could reduce the luciferase activity, while others failed to inhibit the activity (Figure 3A). Once the miR‐200 binding sites were mutated, these miR‐200s failed to reduce the activity (Figure 3A). Second, we performed MS2‐RNA immunoprecipitation (MS2‐RIP) to pulldown miRNAs that directly bind to lncGALM and detected them by qRT‐PCR. Wild‐type lncGALM demonstrated markedly enriched levels of miR‐200s compared to MS2 and lncGALM with miR‐200 binding site mutation (Figure 3B). Finally, we employed RNA affinity pulldown using in vitro transcribed and biotin‐labeled lncGALM to evaluate its direct binding to miR‐200s (Figure 3C). As expected, the wild type of lncGALM displayed strong affinity to bind to miR‐200s; in contrast, lncGALM mutation led to loss of its ability to bind to miR‐200s. However, the expression levels of lncGALM were not affected by the overexpression of miR‐200s (Figure S3D). Taken as a whole, our findings indicate the likely function of lncGALM as a ceRNA to specifically bind to miR‐200s.

It has been reported that ZEB1 and ZEB2 are the target genes of miR‐200s;^24^, ^25^ thus, we questioned whether lncGALM was able to alter ZEB1 and ZEB2 expression through competitive miR‐200s binding. Overexpression of miR‐200s in LiM2‐NOZ cells significantly decreased ZEB1 and ZEB2 expression (Figure S3E and F), but overexpression of lncGALM, rather than mutated lncGALM, in NOZ cells resulted in raised protein and mRNA levels of ZEB1 and ZEB2. Accordingly, these lncGALM‐overexpressing cells demonstrated higher expressions of vimentin and N‐cadherin but suppressed levels of epithelial marker E‐cadherin and ZO‐1. These changes were reversed by the ectopic expression of miR‐200a and miR‐200b (Figure S3G and Figure 3D and E). As expected, downregulation of lncGALM in LiM2‐NOZ cells reduced mRNA and protein levels of ZEB1 and ZEB2, and N‐cadherin and vimentin expression but increased expression of E‐cadherin and ZO‐1. Likewise, inhibition of miR‐200a and miR‐200b restored these effects (Figure S3H and Figure 3F and G). In an attempt to further determine whether these observed effects depend on stabilized gene expression of ZEB1 and ZEB2, we constructed ZEB1 or ZEB2 3′UTR into pmirGLO plasmid. Then pmirGLO, pmirGLO‐ZEB1, or pmirGLO‐ZEB2 was transfected into the respective NOZ and LiM2‐NOZ cells. Overexpression of lncGALM in NOZ cells raised pmirGLO‐ZEB1 and pmirGLO‐ZEB2 luciferase activity compared to mutated lncGALM. Conversely, these findings were abolished via miR‐200a and miR‐200b ectopic activity (Figure 3H). Conversely, downregulation of lncGALM in LiM2‐NOZ cells resulted in suppressed pmirGLO‐ZEB1 and pmirGLO‐ZEB2 luciferase activity, a findings that was reversed upon miR‐200a and miR‐200b inhibition (Figure 3I). Altogether, these results demonstrate that lncGALM promotes EMT by binding to and removing miR‐200s from target ZEB1 and ZEB2 3′UTR, leading to ZEB1 and ZEB2 expression and downstream elevation of N‐cadherin and vimentin.

### 3.4 N‐cadherin mediated the adhesion between GBC cells and LSECs and promoted GBC liver arrest

**FIGURE 4:**
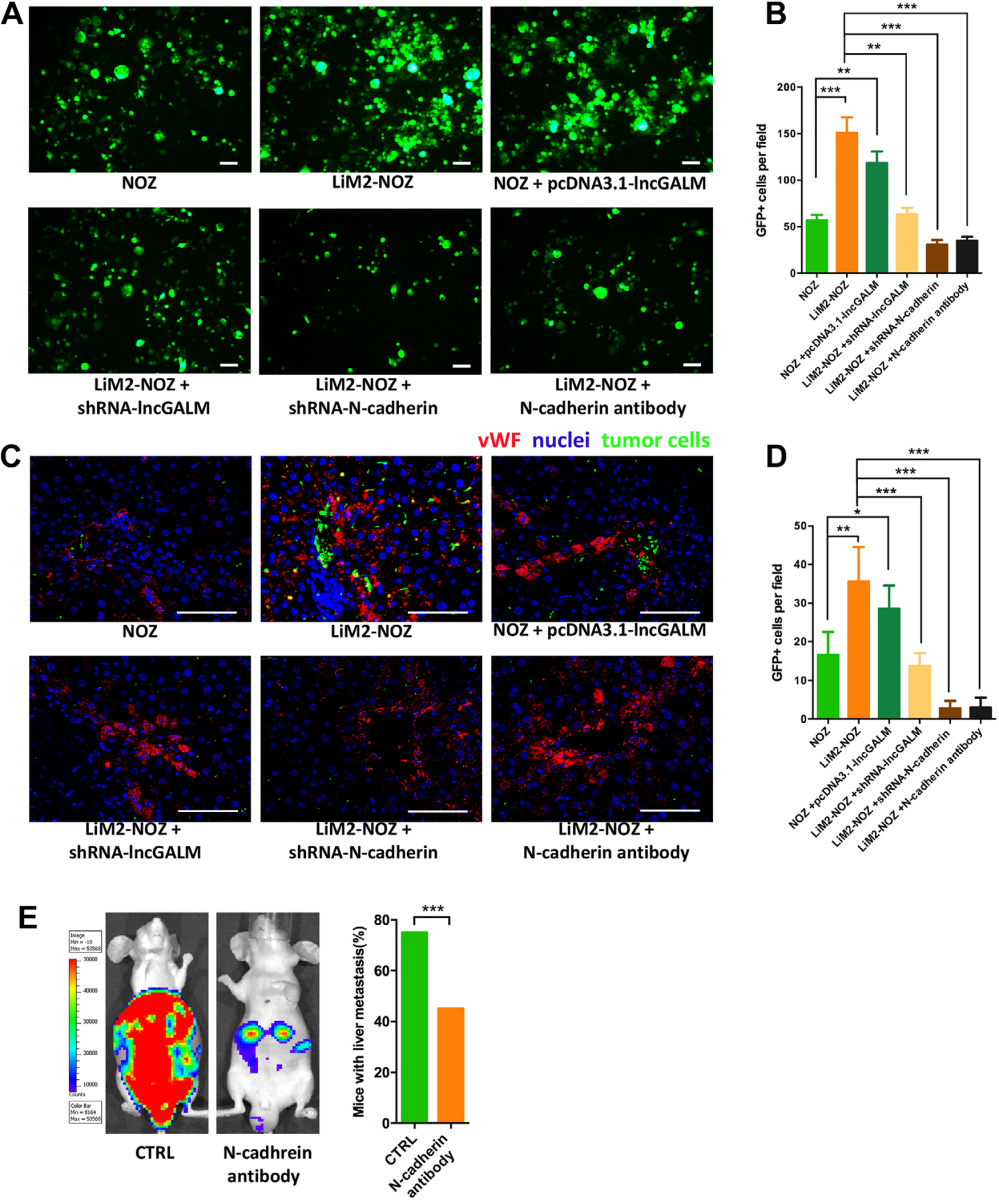
N‐cadherin mediated the adhesion between GBC cells and LSECs. **A,** NOZ cells (labeled with GFP) co‐cultured with LSECs for 6 hours (scale bar, 100μm). **B**, Statistical analysis of the attached GBC cells. **C,** Immunofluorescence of vWF in livers sections 24 hours after the intrasplenic injection of different GBC cells (labeled with GFP), which were attached to the LSECs and arrested at the liver sinusoid (scale bar, 50μm). **D,** Statistical analysis of the arrested GBC cells. **E,** Blockade of N‐cadherin in mice livers and LiM2‐NOZ cells by an N‐cadherin antibody abolished liver metastasis of LiM2‐NOZ cells, and 20 mice were used per cell line. **P < .01, ***P < .001

Tumor cell arrest at a distant organ is initialized at specific regions or tissue, where adhesion molecules expressed by both tumor cells and vascular endothelial cells drive tumor cell migration toward endothelial cells.^20^, ^26^ In an attempt to determine if N‐cadherin mediates GBC arrest via endothelial cell adhesion in the liver, we first isolated primary endothelial cells (LSECs) from normal mouse liver, confirmed with the characterization of endothelial cell marker vWF and CD31 (Figure S4A and B). We found that LSECs strongly expressed N‐cadherin (Figure S4C). Then, we analyzed the potential role of N‐cadherin expression in both GBCs and LSECs in the homophilic interaction during cell adhesion. GFP‐labeled GBC cells were incubated with LSEC monolayer for 6 hours. Three‐fold more LiM2‐NOZ cells than NOZ cells were found in the co‐culture with LSECs (Figure 4A and B). Overexpression of lncGALM in NOZ cells similarly increased cell adhesion, while knockdown of lncGALM in LiM2‐NOZ cells decreased cell adhesion. Strikingly, a neutralizing anti‐N‐cadherin antibody abolished this phenomenon. To further examine the N‐cadherin‐dependent tumor cell arrest in the liver, we performed intrasplenic injections of GFP‐labeled GBC cells in mice. Twenty‐four hours later, mouse livers were removed for immunofluorescence observation. As demonstrated in Figure 4C and D, LiM2‐NOZ cells that were arrested at the liver sinusoids were twofold more than NOZ cells. Overexpression of lncGALM in NOZ cells resulted in increased cell arrest as seen in LiM2‐NOZ cells, whereas knockdown of lncGALM or N‐cadherin in LiM2‐NOZ cells decreased the number of arrested cells. Blocking N‐cadherin by the N‐cadherin antibody impaired adhesion and accumulation of LiM2‐NOZ cells in the liver sinusoids (Figure 4C and D) and subsequently inhibited liver colonization of LiM2‐NOZ cells (Figure 4E). These data demonstrate that N‐cadherin mediates adhesion between GBC cells and LSECs, leading to liver arrest of GBC cells.

### 3.5 LncGALM promoted IL‐1β‐dependent extravasation of GBC cells

**FIGURE 5:**
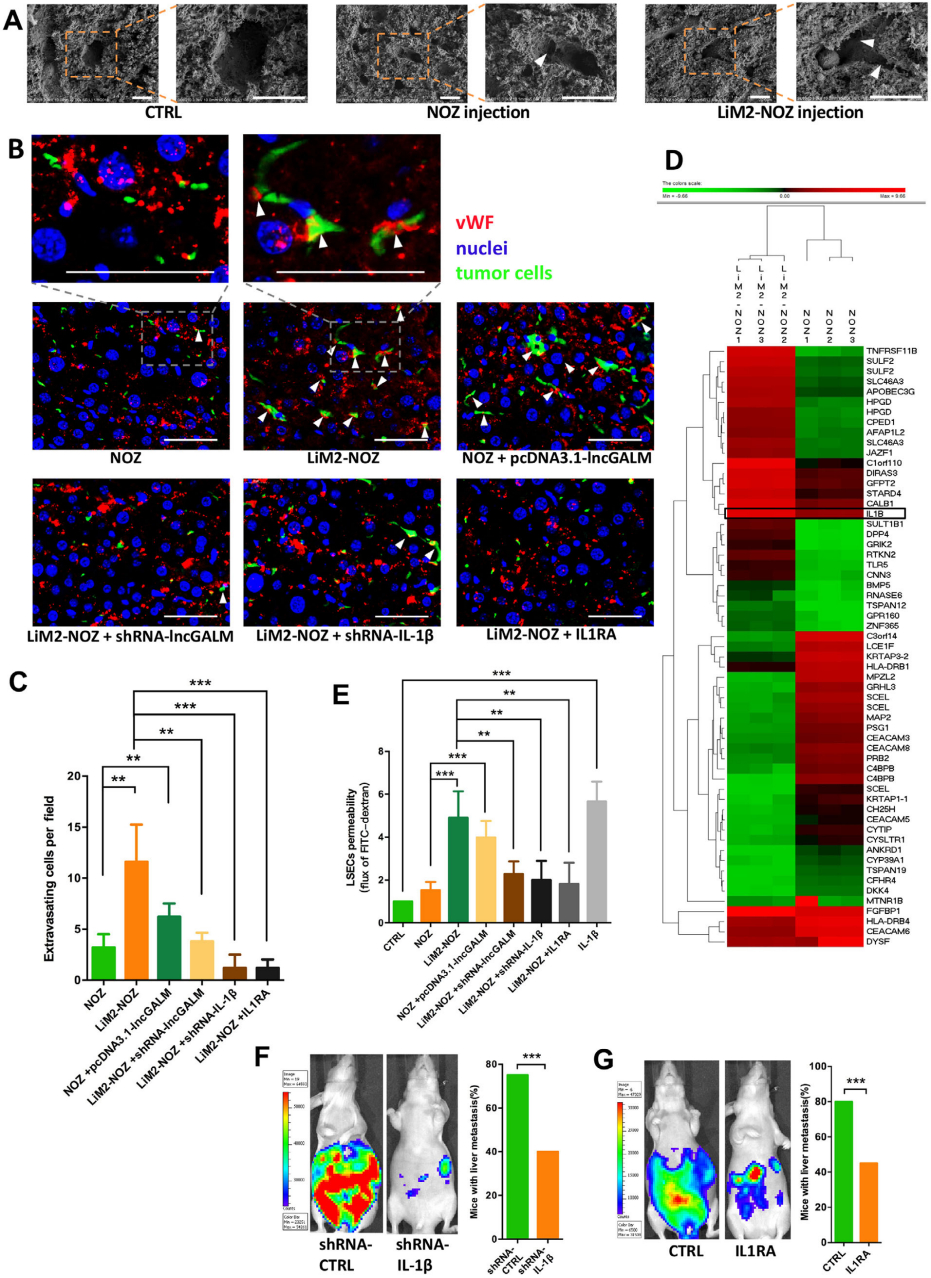
LncGALM promoted extravasation of GBC cells by IL‐1β. **A,** Scanning electron microscope of mouse liver showed the enlarged fenestrae (arrowhead) of liver sinusoid (scale bar, 10μm). **B,** Immunofluorescence of vWF in mouse liver sections injected with different GBC cell clones, the arrowhead showed the extravasating GBC cells (scale bar, 50μm). **C,** Statistical analysis of the extravasating GBC cells. **D**, Hierarchical clustering analysis of the mRNAs that were differentially expressed between NOZ and LiM2‐NOZ cells. **E,** LSECs were seeded on 0.4 μm transwell inserts and treated with conditioned medium from different cell clones. Flux of a 40 kDa fluorescein (FITC)‐dextran tracer was measured. Quantification showed fluorescence counts in the lower chamber normalized to the fresh medium. **F,** Stable knockdown of IL‐1βinhibited LiM2‐NOZ cell liver metastasis, and 20 mice were used per cell line. **G,** Blocking IL‐1β receptor interleukin‐1 receptor 1 (IL1R1) by injecting IL1RA intraperitoneally reduced liver metastasis of LiM2‐NOZ cells, and 20 mice were used per cell line. *P < .05, **P < .01, ***P < .001)

To successfully disseminate to the liver parenchyma and regrowth, GBC cells must break through the barrier that is usually formed by the tight intercellular junctions of LSECs, the process that is known as extravasation.^27^ Forty‐eight hours following intrasplenic injection of GFP‐labeled NOZ and LiM2‐NOZ cells, mouse liver tissues were observed under a scanning electron microscope. In contrast with control sinusoids, NOZ‐penetrated sinusoid wall was characterized by enlarged fenestrae of LSECs, which was even more evident in LiM2‐NOZ tumor models (Figure 5A). To visualize the extravasation of GBCs in the sinusoids, we stained liver sections with an antibody against endothelial cell marker vWF. More extravasating LiM2‐NOZ cells were observed as compared with NOZ cells (Figure 5B and C). Consistent with the earlier observation of cell adhesion, overexpression of lncGALM in NOZ cells increased extravasation, but knockdown of lncGALM in LiM2‐NOZ cells decreased this event (Figure 5B and C).

Next, to validate the hypothesis that impaired endothelial cell permeability is rate limiting for cell extravasation, we selected LSECs for a cell permeability assay in a co‐cultured system with GBCs. We seeded LSECs in the upper chamber of transwells to form a monolayer of cells for 24 hours without adding medium into the lower chamber, then GFP‐labeled GBC cells were loaded on the LSEC monolayer for 48 hours with FBS containing medium in the lower chamber. Twofold more LiM2‐NOZ cells passed through this monolayer barrier than did NOZ cells. Overexpression of lncGALM in NOZ cells also increased cell penetration around 1.5‐fold more than NOZ cells, while knockdown of lncGALM in LiM2‐NOZ cells decreased the cells to those of the control level (Figure S5A and B). Indeed, conditioned medium from LiM2‐NOZ cells resulted in significantly larger gaps between endothelial cells than those from NOZ cells (Figure S5C). The previous studies demonstrated that cytokines secreted by cancer cells play a central role in regulating endothelial cell permeability during cell dissemination^28^, ^29^; thus, we performed gene microarray of NOZ and LiM2‐NOZ cells to identify different expression levels of cytokines that may potentially account for regulation of cell permeability (Figure 5D and Table S4). Interestingly, two cytokines IL‐1β and IL‐15 were upregulated in LiM2‐NOZ cells with eight‐ and sixfold higher than those expressed in NOZ cells. We subsequently evaluated influence of IL‐1β and IL‐15 on LSEC activity. Of note, only IL‐1β decreased viability of LSECs (Figure S5D). Furthermore, the expression levels of IL‐1β correlated with lncGALM expression levels (Figure S5E). As we know, IL‐1β functions through binding to its receptor IL1R and this function could be blocked by an IL1R antagonist (IL1RA).^30^ Accordingly, either knockdown of IL‐1β or blockade of IL1R by an IL1R antagonist (IL1RA) in LiM2‐NOZ cells inhibited cell extravasation (Figure 5B and C) in vivo and the ability to transmigrate through an endothelial monolayer in vitro (Figure S5A and B). Furthermore, the IL‐1β‐dependent endothelial cell permeability induced by LiM2‐NOZ was also evaluated with a 40 kDa FITC‐dextran tracer. IL‐1β conditioned media of LncGALM‐expressing cells increased the permeability of LSECs monolayer; but inhibition of IL‐1β failed to induce high permeability (Figure 5E). Consistent with these in vitro observations, knockdown of IL‐1β and blockade of IL1R in LiM2‐NOZ cells led to inhibition of liver metastasis in nude mice (Figure 5F and G). Based on the above series of experiments, IL‐1β was found to possess an essential role in tumor cell extravasation during liver metastasis.

### 3.6 LncGALM increased IL‐1β mRNA stability and promoted IL‐1β secretion

**FIGURE 6:**
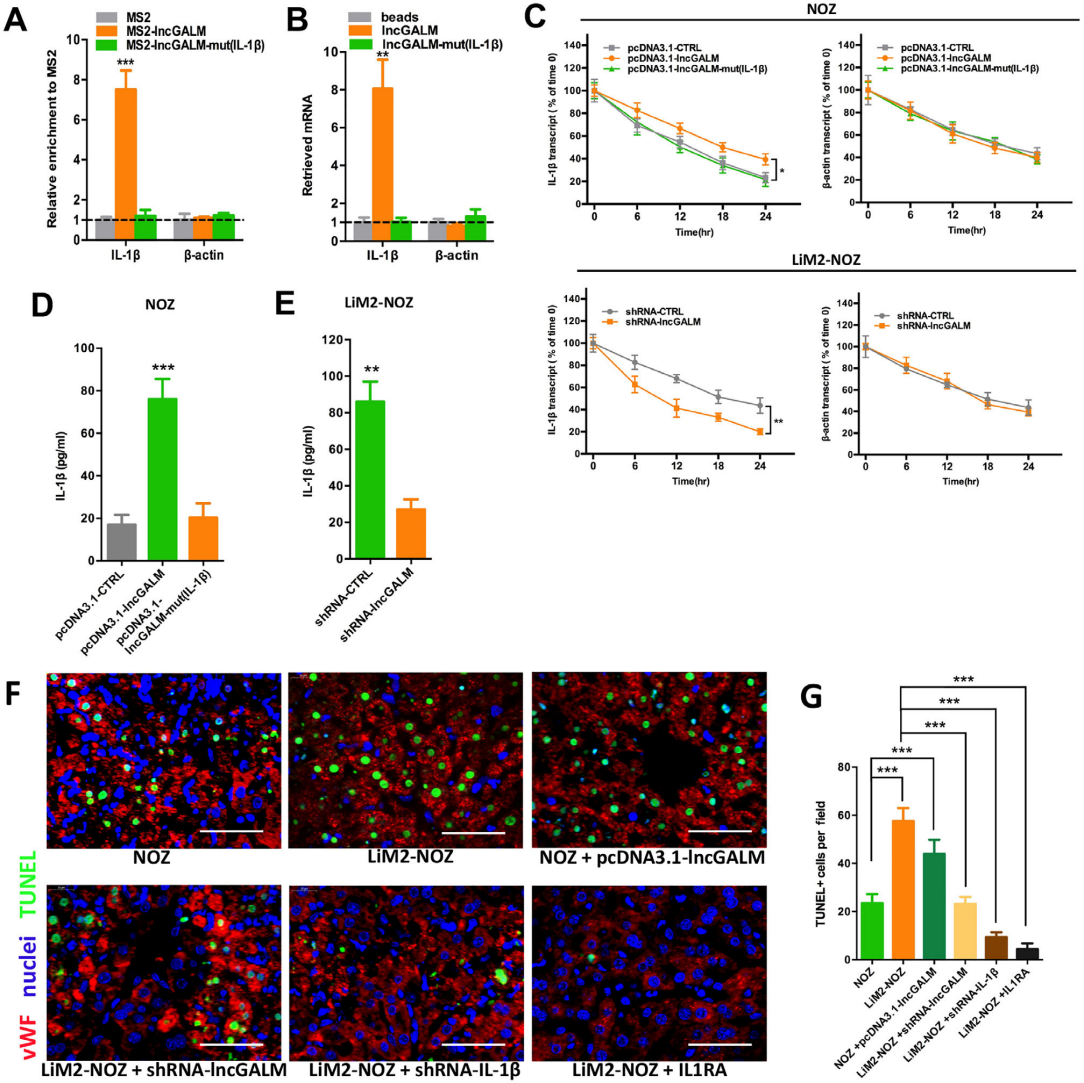
LncGALM increased stability of IL‐1β mRNA and promoted mature IL‐1β secretion. **A,** MS2‐RIP followed by qRT‐PCR was used to test the endogenous binding between lncGALM and IL‐1β. **B,** RNA affinity pulldown followed by qRT‐PCR was used to test the endogenous binding between lncGALM and IL‐1β. **C,** Stability of IL‐1β and β‐actin mRNA relative to that at time 0 was measured by qRT‐PCR after blocking new RNA synthesis with α‐amanitin (50 μM) in the indicated GBC cell clones and normalized to 18S rRNA. **D and E,** Secreted IL‐1β in the indicated NOZ cell (D) and LiM2‐NOZ cell (E) culture supernatants were measured by ELISA. F, TUNEL staining and vWF Immunofluorescence of mouse liver sections to show the apoptosis of LSECs. **G,** The statistical analysis of the TUNEL positive LSECs (scale bar, 50 μm). *P < .05, **P < .01, ***P < .001

We then sought to define the mechanistic link between lncGALM and IL‐1β expression. It is emerging that lncRNAs have the ability to directly interact with mRNAs to increase their stability and thus induce protein expression.^15^, ^31^ To determine whether lncGALM and IL‐1β mRNAs have complementary binding sequences, we performed public data base search in BLAST and interestingly found 16 binding sites with longer than 15 bp present in both sequences (Figure S5F). Then, we performed MS2‐RIP followed qRT‐PCR to identify mRNAs that may directly bind to lncGALM. Abundant IL‐1β mRNA was found to bind to lncGALM relative to vector or β‐actin control mRNAs. Once lncGALM was mutated, IL‐1β failed to bind to lncGALM (Figure 6A). These results were further confirmed by an RNA affinity pulldown assay (Figure 6B). These results suggest that lncGALM can bind to and interact with IL‐1β mRNA directly.

To assess if lncGALM can increase IL‐1β mRNA stability, α‐amanitin was used to treat GBC cells in order to inhibit synthesis of new RNA for 24 hours. Following this treatment period, the degree of degradation of IL‐1β and β‐actin was then measured by qRT‐PCR. As shown in Figure 6C, overexpression of lncGALM in NOZ cells, but not lncGALM‐mut, increased the half‐life period (from *T*_1/2_ = 12 to 18 hours) of IL‐1β mRNA, while knockdown of lncGALM in LiM2‐NOZ cells decreased the half‐life period (from *T*_1/2_ = 18 to 10 hours) of IL‐1β mRNA (Figure 6C). In accordance to findings of the mRNA levels, we found that protein levels of IL‐1β from the supernatant of GBCs overexpressing lncGALM were fourfold higher than control, whereas lncGALM‐mut cells expressed the same level as control cells (Figure 6D). lncGALM gene silence reduced IL‐1β to 30% of the control level (Figure 6E). In conclusion, our findings indicate that lncGALM may bind to and stabilize IL‐1β mRNA, leading to increased protein expression of IL‐1β that augments LSEC permeability.

### 3.7 IL‐1β induced LSEC apoptosis to facilitate GBC cells extravasation

We further evaluated the possibility of IL‐1β in inducing endothelial cell apoptosis that occurs in the late phase of extravasation of tumor cells, because IL‐1β is known to promote cell death.^32^ To test this hypothesis, we analyzed cell death using a TUNEL staining assay in mouse liver sections 48 hours after GFP‐labeled GBC cells were administrated via intrasplenic injection. LiM2‐NOZ tumors exhibited approximately 2.8‐fold more apoptosis in endothelial cell area than NOZ tumors (Figure 6F, G), leaving largely spacious sinusoids in the absence of nuclei. Like LiM2‐NOZ tumors, tumors overexpressing lncGALM induced twofold greater cell death than controls, while tumors with knockdown of lncGALM decreased the apoptosis levels to those of the control level (Figure 6F and G). Strikingly, knockdown of IL‐1β and block IL‐1R could eliminate cell apoptosis (Figure 6F and G), suggesting that IL‐1β acts as a strong death‐promoting factor in destroying intact wall structure of sinusoids during tumor cell metastasis. These data suggest that IL‐1β induced LSEC apoptosis to facilitate the extravasation of GBC cells.

## 4 DISCUSSION

The liver is the most common site for GBC distant metastasis, the event that eventually leads to terminal disease.^3^, ^4^ Most of GBC patients with liver metastasis, unfortunately, are inoperable, and typically receive palliative chemotherapy and radiotherapy. However, this nonsurgical intervention is not a curative method. Therapeutic efficacy relies strongly on specific targeting of key molecules that regulates the malignant transformation of GBC cells during metastasis. In fact, our current knowledge about metastasis‐driven molecules in GBC is minimal; thereby, unveiling novel factors is of paramount importance in cancer therapy. Here, we focused on lncRNA alterations in cancer samples from primary GBC cancer and metastatic cancer. In addition, to solely establish the key role of new lncRNAs in GBC metastasis, we simultaneously created a metastatic mouse model followed by an analysis of the differential lncRNAs profiles between primary and metastatic cancer in both systems. As a result, we identified lncGALM as a metastasis‐associated lncRNA that commits GBC to dissemination in the liver. This finding may provide a new biomarker for GBC liver metastasis and a target potential for therapy.

It is emerging that a number of pathological and biological processes are mediated by lncRNAs, which take part in diverse regulatory mechanisms, one of which involve functioning as endogenous RNAs (ceRNAs) sponges to bind to and remove miRNA from target genes.^21^, ^22^, ^23^ In this action model, lncRNAs harboring the same miRNA response element as the target mRNA were found to be able to influence the expression levels of mRNA through competitive sequestration to shared miRNAs. In cancer, several lncRNAs were reported to function as ceRNAs that trigger tumor progression and drug resistance.^15^, ^22^ In concert with these reports, we hypothesize that lncGALM functions as a ceRNA that can specifically bind to and sequester miR‐200s, which interact with and inhibit ZEB1 and ZEB2 mRNA expression. As essential transcription factors capable of regulating EMT, ZEB1 and ZEB2 induced by lncGALM promoted GBC to undergo EMT. Of note, the EMT‐associated gene activity and protein expression were fully abolished by overexpression of miR‐200a and miR‐200b, both of which represent members of the miR‐200 family.

Tumor metastasis is characterized as sequelae of malignant tumor cells, a process that develops as a result of a dynamic interaction between cancer cells and vascular tissue, including initial migration, adhesion, and penetration through (extravasation) vascular endothelial cells, and finally residing at a foreign region to regrow. There is a wealth of research evidence demonstrating that upregulated N‐cadherin renders cancer cells more adhesive and invasive in cancer dissemination.^33^, ^34^ Consistent with this notion, we found that N‐cadherin induced by lncGALM prompted GBC migration toward and adhesion with endothelial cells that are rich in the liver sinusoidal. Indeed, tumor cells highly expressing lncGALM displayed a fibroblastic phenotype with elevated levels of N‐cadherin, thus facilitating cell migration to and interaction with endothelial cells.

Extravasation is a critical process following tumor cell arrest in distant organs where tumor cells penetrate through vascular endothelial cells into the tissue parenchyma in order to disseminate.^35^ To circumvent the physical barrier that is predominantly formed from endothelial cell‐to‐cell contacts, aggressive tumor cells must secrete a large number of active factors sufficient to impair these obstacles and eventually increase vascular permeability.^7^, ^29^ For example, VEGF, PLGF, MMP‐10, MMP‐3, Angpt2, and PTHLH released from tumor cells in various types of tumors are capable of triggering pulmonary endothelium hyper‐permeability, thereby accelerating extravasation of tumor cells.^29^, ^36^, ^37^, ^38^ In this study, we identified elevated IL‐1β and IL‐15 transcript levels in lncGALM‐expressing LiM2‐NOZ cells, and found that IL‐1β harbors multiple complementary binding sites of lncGALM and lncGALM binding, which led to IL‐1β mRNA stabilization. IL‐1β secreted from GBC cells played a central role in the cell extravasation to the liver, as IL‐1β induced endothelial cell apoptosis and permeability, giving rise to cellular fenestration favorable for tumor cell extravasation. We also noted the possibility in vivo that other cells, except tumor cells and LSECs, may induce IL‐1β expression such as kupffer cells and stellate cells, which constitute a positive loop to enhance sinusoidal disruption and facilitate liver metastasis of GBC cells. This may partially explain why liver is the most common target organ of GBC cells distant metastasis.

## 5 CONCLUSIONS

**FIGURE 7:**
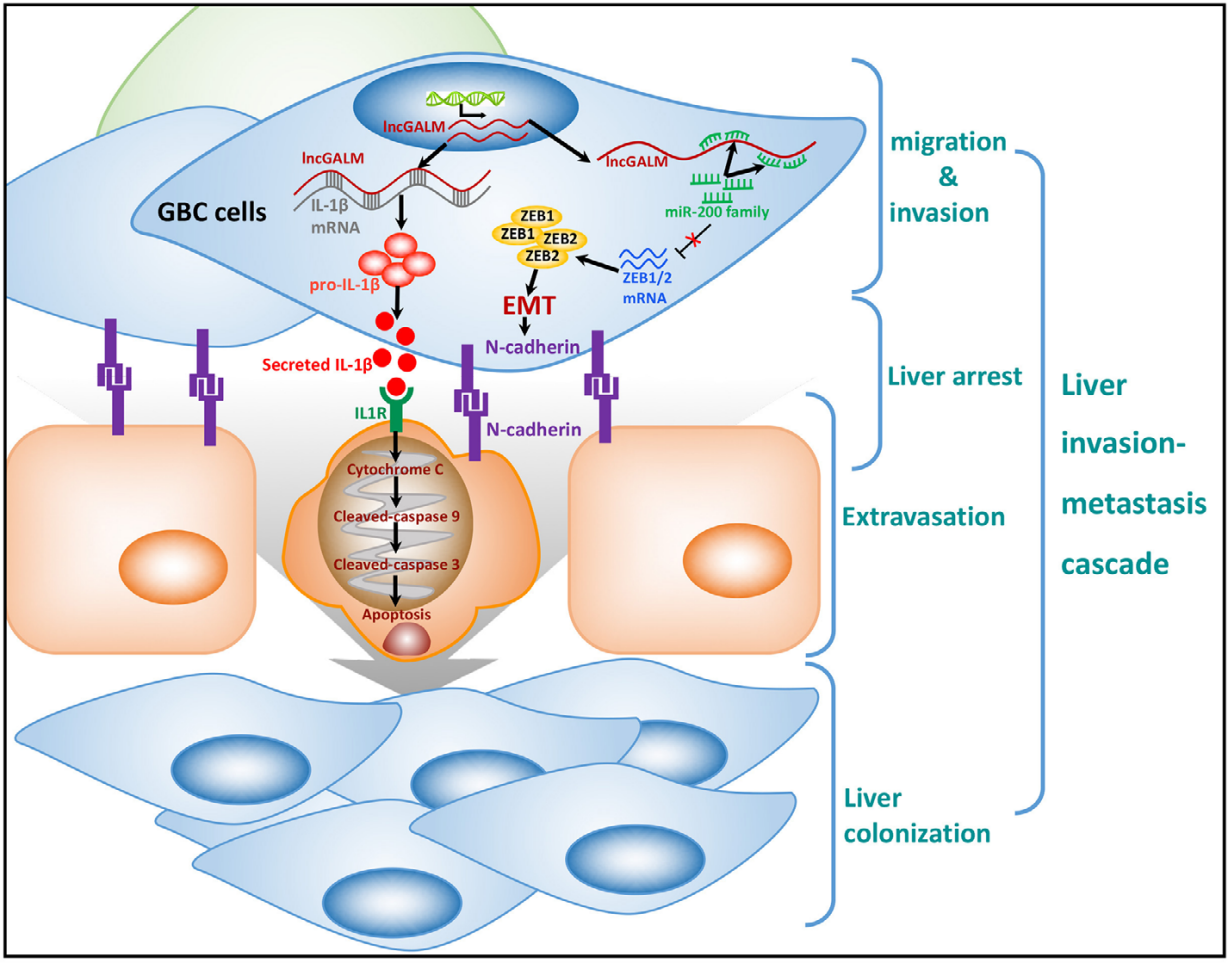
Schematic diagram of lncGALM functions to promote liver metastasis in GBC cells

Collectively, we reported a novel lncRNA‐lncGALM that promotes GBC liver metastasis through regulation of multiple target genes that coordinately contribute to tumor cell migration, arrest, and extravasation in the liver (Figure 7). These findings have pointed to lncGALM not only as a likely biomarker for liver metastasis, but also a promising therapeutic target for GBC.

## CONFLICT OF INTERESTS

The authors declare no potential conflict of interest.

## ETHICS APPROVAL AND CONSENT TO PARTICIPATE

The human study was approved by the Institutional Ethical Board of Xinhua Hospital and informed consent was obtained from all patients with GBC prior to enrollment of the study. All of the animal studies were approved by the Institutional Animal Care and Use Committee of Xinhua Hospital, Shanghai Jiao Tong University School of Medicine, Shanghai, China. All the animal experiments were performed according to our institution's guidelines for the use of laboratory animals.

## AUTHOR CONTRIBUTIONS

Yingbin Liu, Xiangsong Wu and Jian Zhu conceived and directed the study. Huaifeng Li, Yunping Hu and Yingbin Liu contributed to the project design. Huaifeng Li, Yunpeng Jin, Yidi Zhu, and Yang Yang performed the experiments. Huaifeng Li, Fatao Liu, Yuanyan Ye, Shanshan Xiang, and Xiaoling Song analyzed the bioinformatics data. Guoqiang Li, Yijian Zhang, Lin Jiang, Wen Huang contributed samples, data, and comments on the manuscript. Huaifeng Li analyzed and interpreted the data. Yuan Gao, Yajuan Hao, and Jinhui Zhu contributed reagents, materials, and/or analysis tools. Huaifeng Li wrote the manuscript.

## Supporting information

Supporting information

Supporting information

## Data Availability

RNA sequencing data are deposited at the Gene Expression Omnibus database with the accession Number GSE132223. Microarray data are deposited at the Gene Expression Omnibus database with the accession Number GSE106671.
