# Supplementary material for: Long noncoding RNA lncGALM increases risk of liver metastasis in gallbladder cancer through facilitating N‐cadherin and IL‐1β‐dependent liver arrest and tumor extravasation
Source: Clin Transl Med. 2020 Nov 10;10(7):e201. doi: 10.1002/ctm2.201 (PMC7653798; doi:10.1002/ctm2.201)
Supplement: Supplementary file 1 — Supporting information [file CTM2-10-e201-s001.doc]

**Supplementary Figures**

**
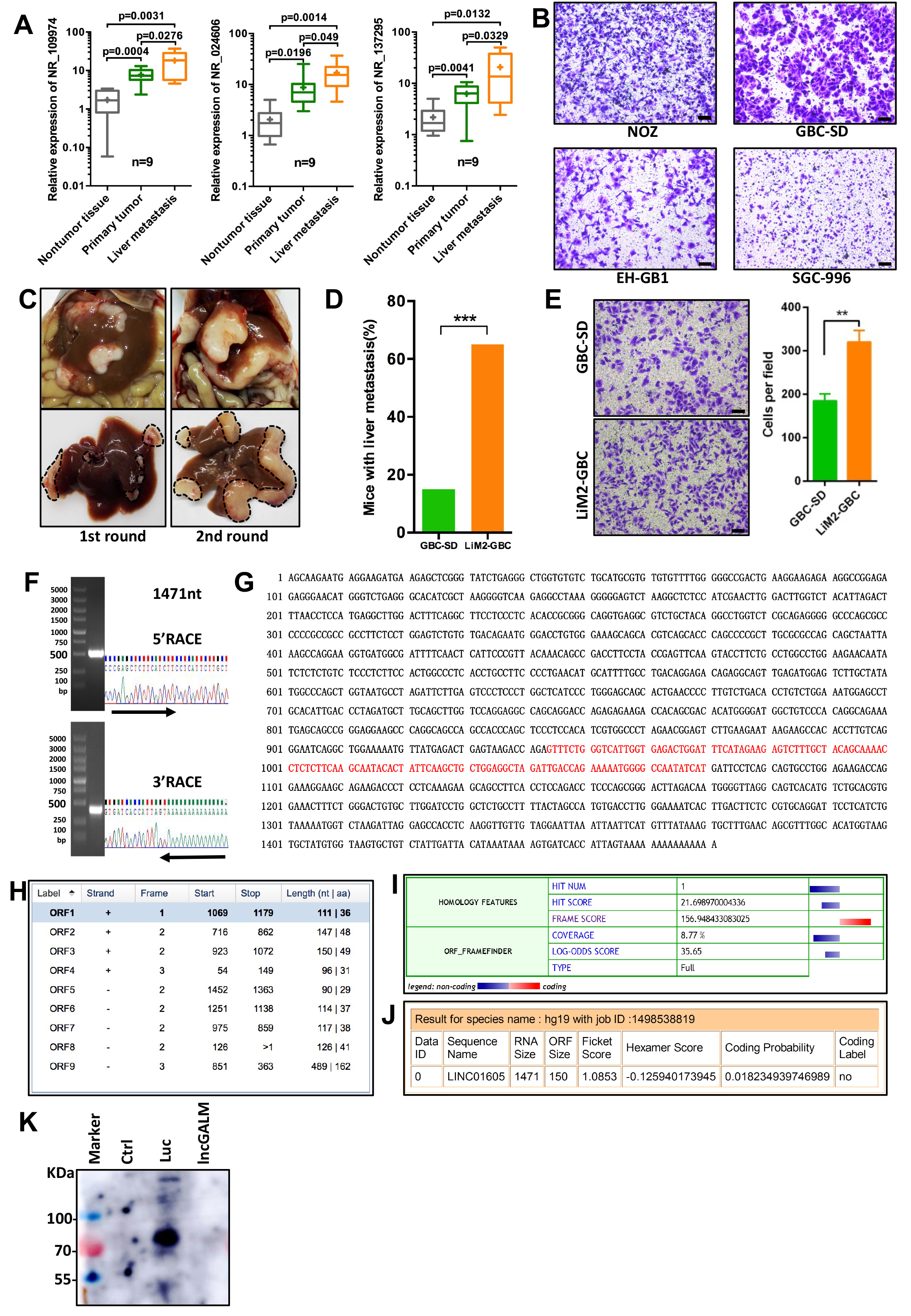
**

**Figure S1. The selection process of lncGALM, which has no protein-coding potential.** (A) The expression levels of 3 lncRNAs in 9 GBC patients’ samples. (B) The migration ability of four GBC cell lines assessed by transwell assay; 2×10^4^ cells were used for each cell line and cultured for 18 hours. (C) GBC-SD and LiM2-GBC cell liver metastasis lesions. (D) Liver metastasis rate of GBC-SD and LiM2-GBC cells, 20 mice were used per cell line, two-tailed Fisher’s exact test was used to calculate statistical signiﬁcance. (E) The migration ability of GBC-SD and LiM2-GBC cells assessed by transwell assay in three independent experiments. (F and G) The whole sequence of lncGALM determined by RACE. (H, I and J) Coding potential of lncGALM predicted by ORF Finder, CPC and CPAT. (K) In vitro translation assay using luciferase (Luc) as a positive control. (scale bar, 100 μm). (**P<0.01, ***P<0.001).


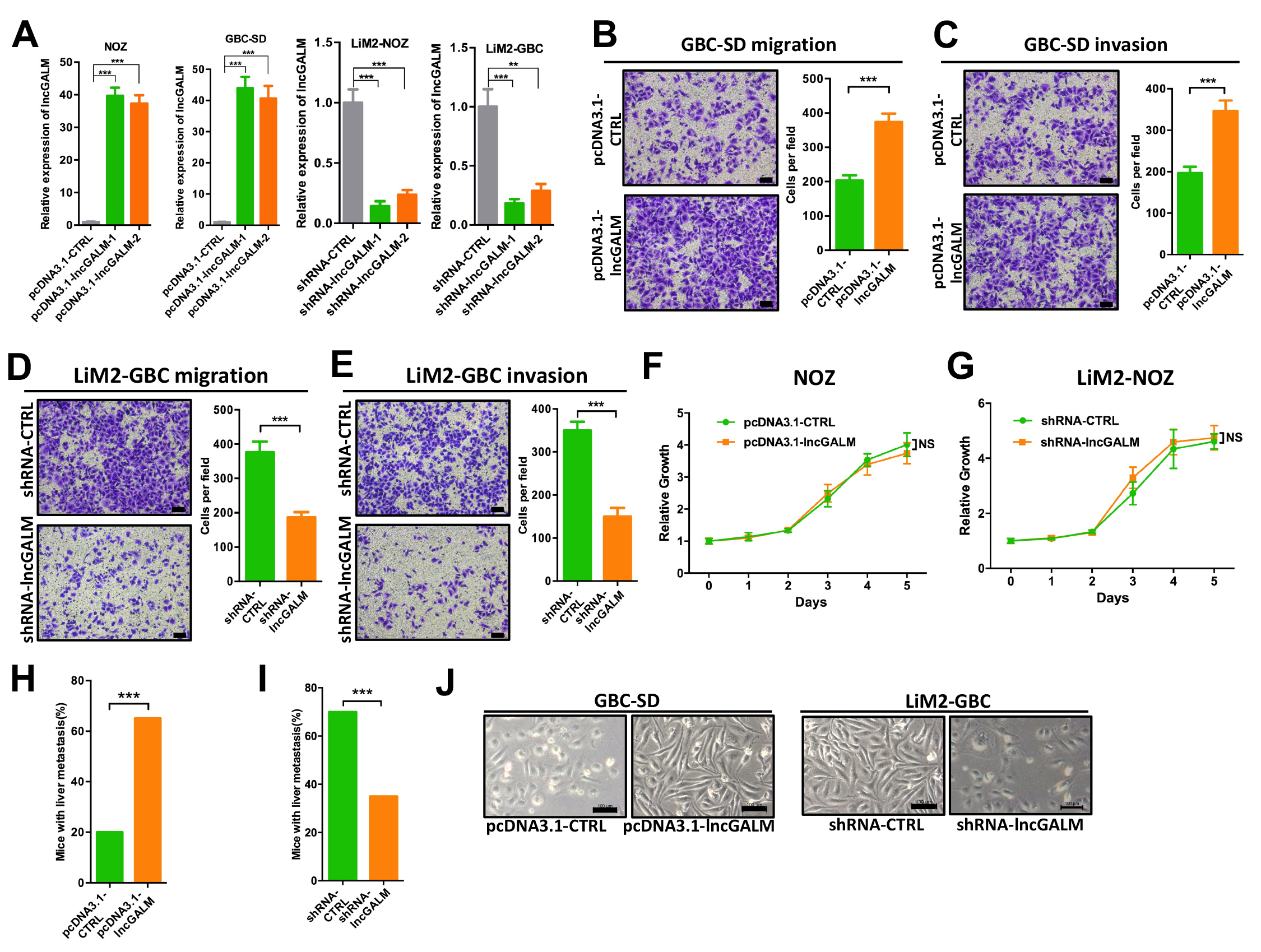


**Figure S2. LncGALM promoted metastasis in GBC-SD cells.** (A) Overexpression efficiency of lncGALM in NOZ and GBC-SD cells by two independent plasmids and knockdown efficiency of lncGALM in LiM2-NOZ and LiM2-GBC cells by two shRNA; we chose pcDNA3.1-lncGALM-1 to overexpress and shRNA-lncGALM-1 to knock down lncGALM in the following experiments. (B and C) LncGALM overexpression significantly increased the migration and invasion ability of GBC-SD cells *in vitro*. (D and E) LncGALM knockdown significantly decreased the migration and invasion ability of LiM2-GBC cells *in vitro*. (F and G) The proliferation ability of NOZ and LiM2-NOZ cells after lncGALM overexpression and knockdown was determined by CCK-8 assay. (H) LncGALM overexpression promoted GBC-SD cell liver metastasis, 20 mice were used per cell line. (I) LncGALM knockdown suppressed LiM2-GBC cell liver metastasis, 20 mice were used per cell line. (J) LncGALM overexpression induced mesenchymal-like morphological features in GBC-SD cells (upper panel), while lncGALM knockdown reversed this change in LiM2-GBC cells (lower panel). (scale bar, 100 μm). (**P<0.01, ***P<0.001).


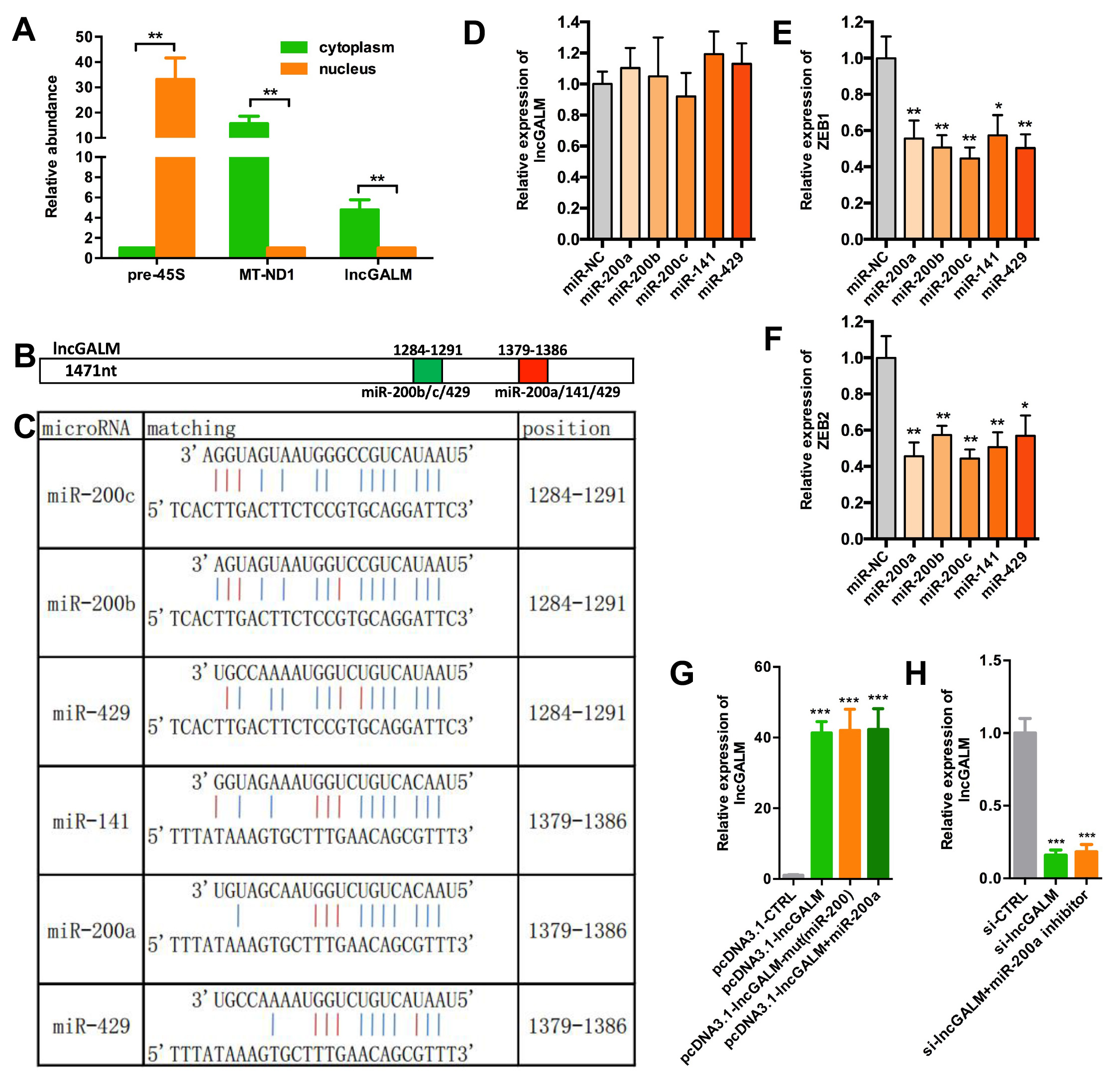


**Figure S3. The binding sites between lncGALM and miR-200 family members.** (A) Subcellular location of lncGALM tested by separating cytoplasmic and nuclear RNA, followed by qRT-PCR. Pre-45S rRNA was mainly located in the nucleus, and MT-ND1 was mainly located in the cytoplasm. (B and C) The binding sites of lncGALM and miR-200 family members. (D, E, F) LncGALM (D), ZEB1 (E) and ZEB2 (F) expression levels after overexpression of miR-200 family members in LiM2-NOZ cells. (G) Overexpression efficiency of lncGALM in different NOZ cell clones. (H) Knockdown efficiency of lncGALM in different LiM2-NOZ cell clones. (*P<0.05, **P<0.01, ***P<0.001).


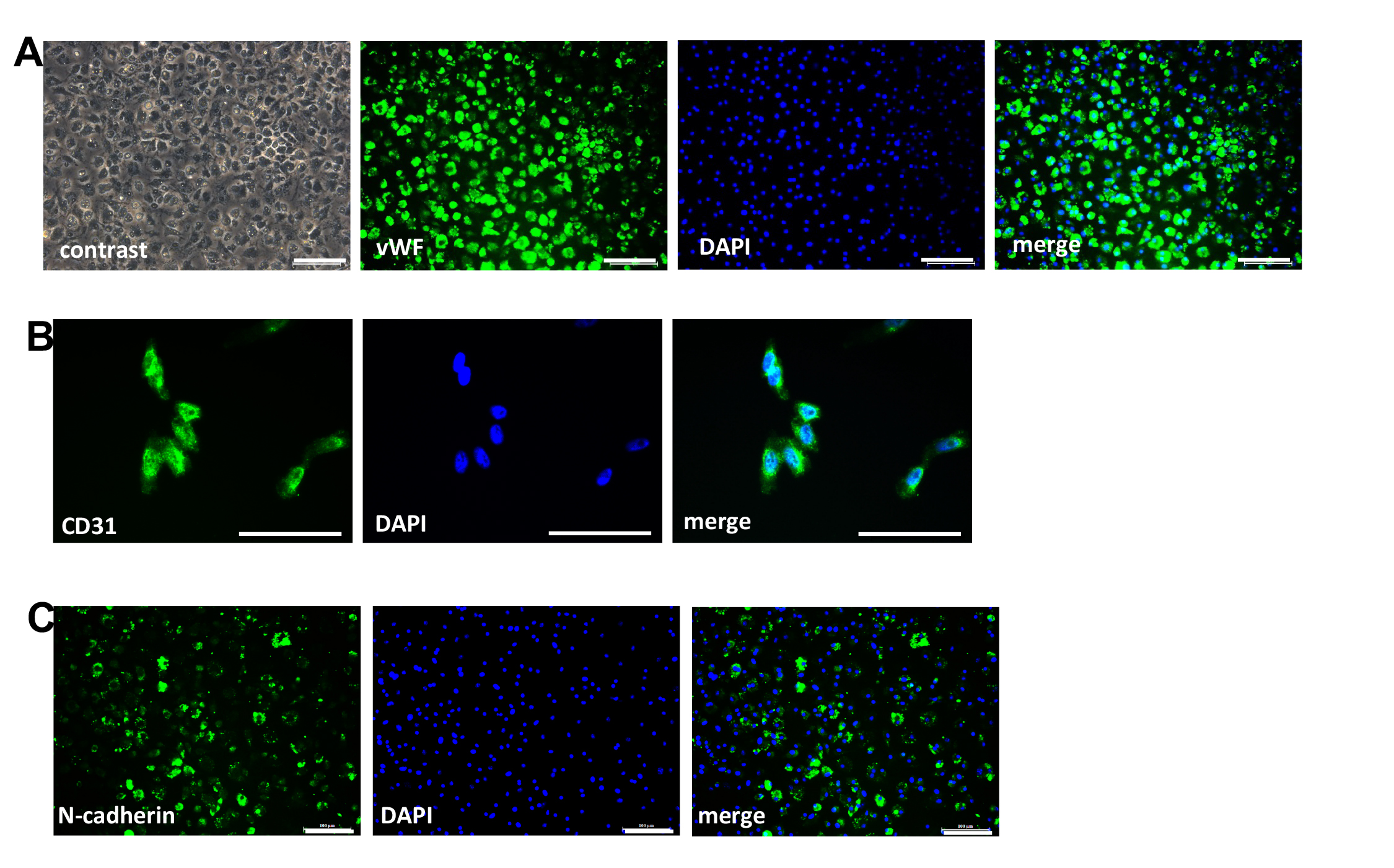


**Figure S4.** **The isolation of mouse LSECs.** (A), LSECs were isolated from mouse liver tissue and confirmed by vWF immunoﬂuorescence. (B), LSECs isolated from mouse liver tissue were confirmed by CD31 immunoﬂuorescence. (C), N-cadherin was abundantly expressed in LSECs. (scale bar, 100 μm)


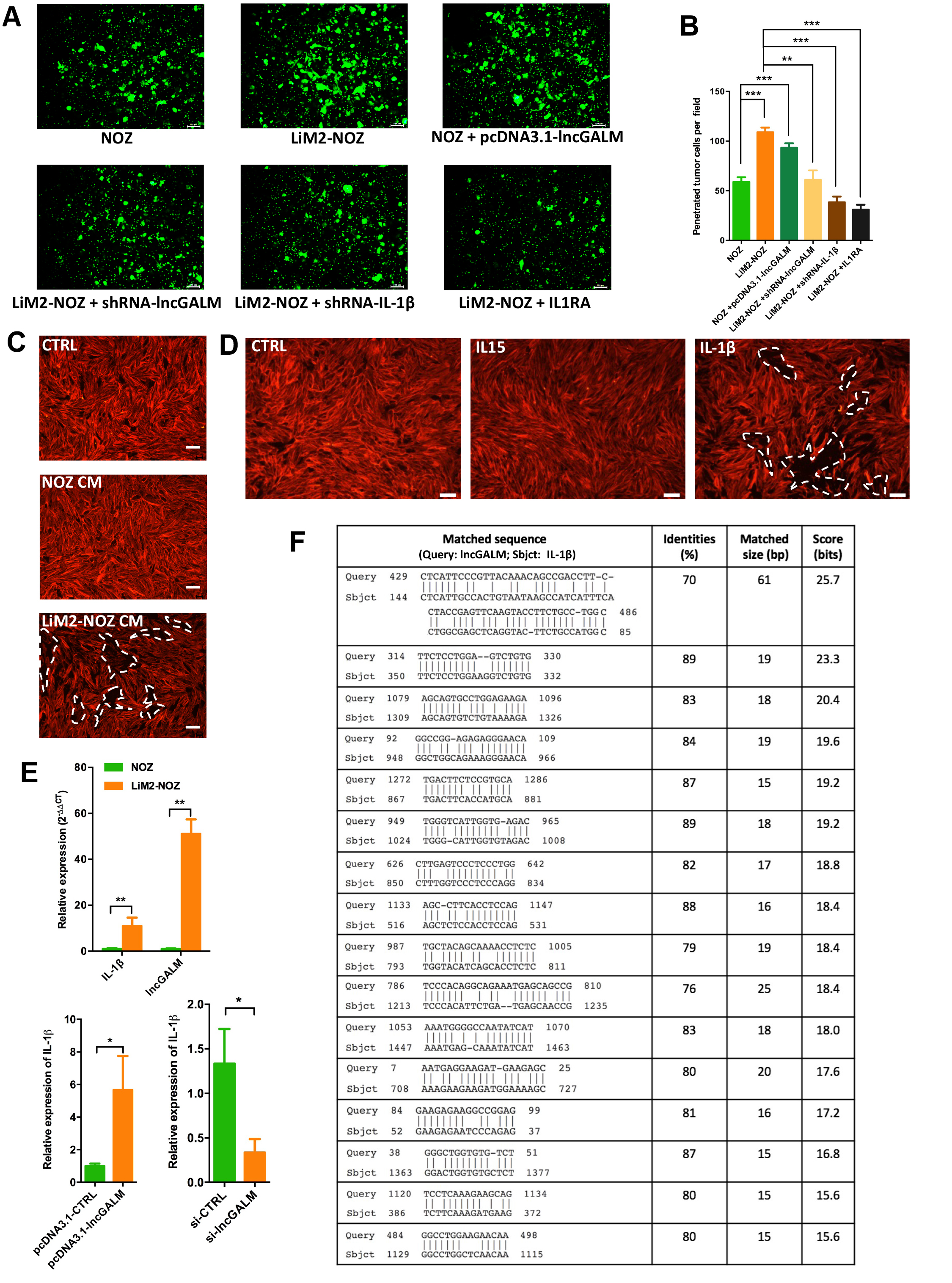


**Figure S5. LncGALM induced LSEC apoptosis by IL-1β.** (A) GFP-labeled GBC cells that pass through the LSEC monolayer (scale bar, 100 μm). (B) The statistical analysis of the trans-monolayer GBC cells. (C) LSECs cultured with NOZ and LiM2-NOZ cell culture supernatants for 48 hours and labeled with phalloidin. (D) LSECs treated with IL-1β and IL-15 for 48 hours and labeled with phalloidin. (E) The mRNA levels of IL-1β were in agreement with the lncGALM levels. (F) In all, 16 binding sites longer than 15 bp between the lncGALM sequence and the IL-1β mRNA sequence were predicted using BLAST (NCBI). (scale bar, 100 μm). (*P<0.05, **P<0.01).
